# Defining the proteomic landscape of cultured macrophages and their polarization continuum

**DOI:** 10.1111/imcb.12687

**Published:** 2023-09-11

**Authors:** Tiah CL Oates, Pedro L Moura, Stephen Cross, Kiren Roberts, Holly E Baum, Katy L Haydn‐Smith, Marieangela C Wilson, Kate J Heesom, Charlotte E Severn, Ashley M Toye

**Affiliations:** ^1^ School of Biochemistry, Biomedical Sciences Building University of Bristol Bristol UK; ^2^ National Institute for Health Research Blood and Transplant Research Unit (NIHR BTRU) in Red Blood Cell Products University of Bristol Bristol UK; ^3^ Center for Haematology and Regenerative Medicine, Department of Medicine (MedH) Karolinska Institutet Huddinge Sweden; ^4^ University of Bristol Bristol UK; ^5^ Max Planck Bristol Centre for Minimal Biology, School of Chemistry University of Bristol Bristol UK; ^6^ Proteomics Facility, Biomedical Sciences Building University of Bristol Bristol UK

**Keywords:** dexamethasone, macrophage, macrophage subtype, polarization, proteomics

## Abstract

Macrophages have previously been characterized based on phenotypical and functional differences into suggested simplified subtypes of MØ, M1, M2a and M2c. These macrophage subtypes can be generated in a well‐established primary monocyte culture model that produces cells expressing accepted subtype surface markers. To determine how these subtypes retain functional similarities and better understand their formation, we generated all four subtypes from the same donors. Comparative whole‐cell proteomics confirmed that four distinct macrophage subtypes could be induced from the same donor material, with > 50% of 5435 identified proteins being significantly altered in abundance between subtypes. Functional assessment highlighted that these distinct protein expression profiles are primed to enable specific cell functions, indicating that this shifting proteome is predictive of meaningful changes in cell characteristics. Importantly, the 2552 proteins remained consistent in abundance across all macrophage subtypes examined, demonstrating maintenance of a stable core proteome that likely enables swift polarity changes. We next explored the cross‐polarization capabilities of preactivated M1 macrophages treated with dexamethasone. Importantly, these treated cells undergo a partial repolarization toward the M2c surface markers but still retain the M1 functional phenotype. Our investigation of polarized macrophage subtypes therefore provides evidence of a sliding scale of macrophage functionality, with these data sets providing a valuable benchmark resource for further studies of macrophage polarity, with relevance for cell therapy development and drug discovery.

## Graphical abstract

In this study, we have explored an *in vitro* culture system that enables the *ex vivo* generation of four proteomically distinct macrophage subtypes from single donors. These macrophage subtypes maintain a set of commonly expressed proteins that facilitate repolarization, as evidenced by treatment of M1 macrophages with dexamethasone, which induces cross‐polarization toward M2c surface markers while retaining the M1 functional phenotype.

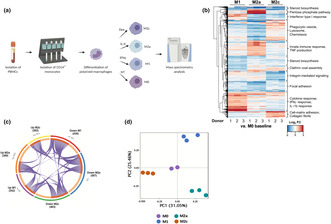

## INTRODUCTION

Macrophages are specialized immune cells with broad functional heterogeneity. In addition to the detection and destruction of bacteria and viruses, macrophages produce a cocktail of cytokines stimulating a variety of immuno‐pathologies. An important feature of macrophages is their ability to exhibit plasticity in their phenotype, differentiating into pro‐ and anti‐inflammatory subtypes in response to the local environment.^1^ T helper cells exhibit distinct cytokine secretion profiles leading to the generation of macrophages with unique phenotypes. Type 1 T helper cells induce the proinflammatory “classically activated” macrophages referred to as M1, while Type 2 T helper cells induce macrophages that are “alternatively activated.”^2^, ^3^ M2 macrophages are further subdivided into M2a, M2b, M2c and M2d subtypes based on applied stimuli and the transcriptional changes induced.^4^ M2a macrophages are “alternatively activated” induced by interleukin‐4, modulating the proinflammatory response and wound healing.^5^ The M2c phenotype induced by glucocorticoids are considered anti‐inflammatory “deactivated” macrophages with a role in tissue remodeling; additionally, they are central to the erythroblastic island.^1^, ^6^, ^7^ Macrophage subtypes are generally identified based on cell surface markers. M1 macrophages being defined by high expression of CD14, CD80, CD86 and CD16; M2a macrophages typically have lower expression of CD14 and CD16 and higher expression of CD206 and M2c macrophages comparatively express high levels of CD14, CD169, CD163 and CD206.^4^, ^7^, ^8^, ^9^ While this M1/M2 classification provides a useful framework to classify macrophage function, more recent studies have demonstrated that macrophages retain plasticity once polarized, a state that is influenced by signaling cytokines as well as the local tissue environment.^10^, ^11^ Therefore, we aim to further understand macrophages beyond the dichotomy of classically and alternatively activated macrophages and to explore the boundaries of macrophage polarity.

In this study, we have comprehensively characterized four human *ex vivo* cultured macrophage subtypes, all generated from the same originating donors, with a focus on semiquantitative proteomics (total and surfaceome) and functionality. Importantly, we demonstrate distinct expression patterns of proteins between the subtypes, which are dependent on the macrophage phenotype and function. Our approach of comparing subtypes using whole cell proteomics demonstrates that macrophages maintain a core proteome across subtypes, which likely facilitates retention of macrophage identity and enables repolarization between subsets. To explore alterations that occur during repolarization, we also investigated the effects of dexamethasone exposure on the inflammatory M1 subtype, characterizing the effect of application of this steroid on proinflammatory macrophage polarization, showing that the glucocorticoid can induce a macrophage with a combined phenotype from two subtypes. This illustrates that macrophages are essentially a cell type that exists on a continuum of phenotypes, enabling more heterogeneity to be induced. Through this detailed dissection of macrophage biology, we improve the understanding of macrophage polarity, providing novel data sets relevant to the macrophage research community.

## RESULTS

### Distinct macrophage subtypes are differentiated from human cultured macrophages

**Figure 1:**
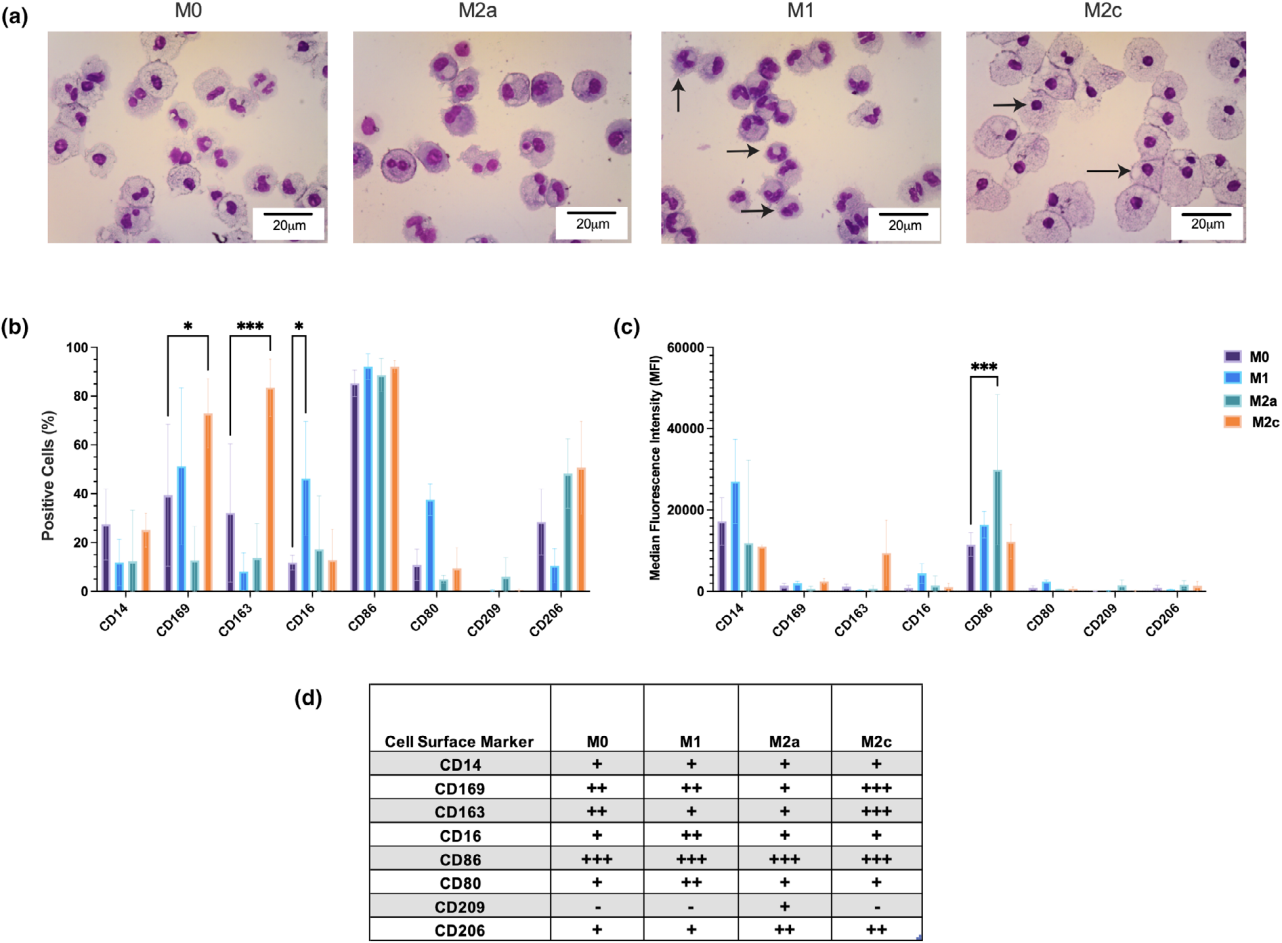
Human cultured polarized macrophage subtypes differ in cell surface marker expression and morphology. **(a)** Representative cytospins at day 7 from each of the subtypes, MØ control, M1, M2a and M2c stained with May–Grünwald's and Giemsa's stain. Scale bars are 20 μm; images are representative of *N* = 3. **(b)** Flow cytometry analysis summary of cell surface expression for key macrophage markers at day 7 of *ex vivo* culture across four different subtypes; CD14, CD169, CD163, CD16, CD86, CD80, CD209 and CD206. *N* = 3 with a minimum of 10 000 events per sample. **(c)** Median fluorescence intensity of flow cytometry markers tested in **b**. All flow cytometry experiments show the mean and standard deviation for the data (*N* = 3, ns *P* ≥ 0.05), with a minimum of 10 000 events per sample and significance tested using a one‐way ANOVA followed by a Tukey's multiple comparison test, comparing each subtype. **(d)** Summary of cell surface marker expression in the four cultured macrophage subtypes assessed by flow cytometry. Expression presented as a scale. *P < 0.05; ***P < 0.001.

CD14^+^ monocytes isolated from healthy donor peripheral blood mononuclear cells were utilized to culture four different macrophage populations; MØ control macrophages, proinflammatory M1 stimulated with interferon gamma (IFNγ), alternatively activated M2a macrophage stimulated with interleukin‐4 and deactivated M2c macrophages stimulated with dexamethasone.^12^, ^13^, ^14^ To confirm that four distinct macrophage subtypes could be produced from a single originating donor, we first assessed cell morphology utilizing cytocentrifugation and known surface markers. It was observed that classically activated M1 macrophages display lobed nuclei and cell projections in the form of filipodia, while the nuclei of M2c deactivated macrophages were more condensed and the cells larger (indicated by arrows in Figure 1a). Next, cell surface expression of the four *ex vivo* generated macrophage subtypes was explored using flow cytometry. The expression of CD169 and CD163 was significantly increased in macrophages polarized toward the M2c state compared with MØ cells. In M1 cells the expression of CD16 was significantly increased compared with the MØ control, and these cells had the highest expression of CD80. CD209 was not expressed in any macrophage population except M2a macrophages. Median fluorescence intensity–highlighted surface expression levels of CD14 were significantly increased in M1 macrophages compared with both M2a and M2c macrophages, and CD86 was significantly increased in M2a macrophages in comparison to macrophages in other states (Figure 1b–d and Supplementary figure 1).

### Whole‐cell quantitative proteomics reveal the extended scale of macrophage subtype plasticity

**Figure 2:**
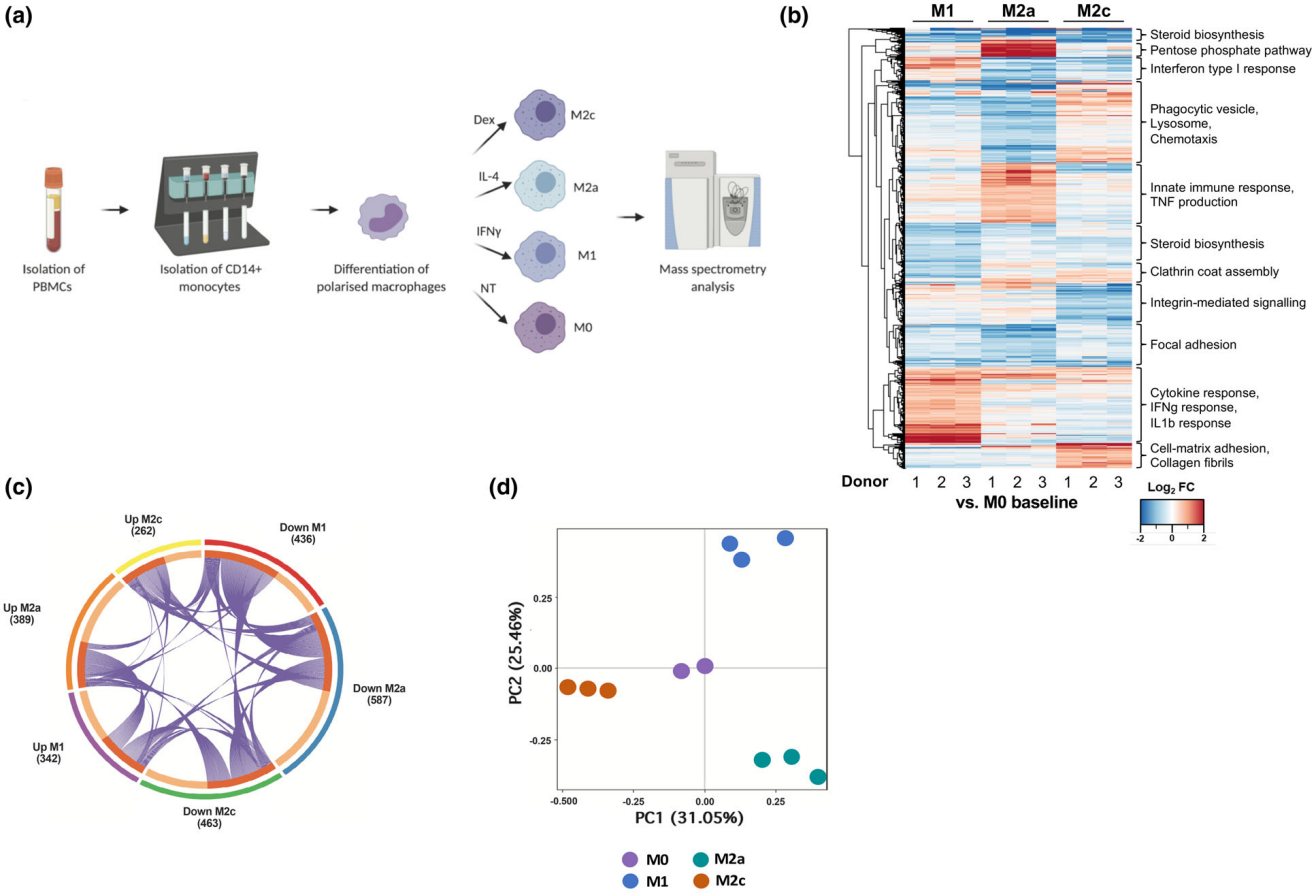
Semiquantitative tandem mass tagged (TMT) proteomics highlights the significant proteomic changes underlying macrophage polarization. **(a)** Experimental design for TMT proteomic analysis. CD14^+^ cells were isolated from donated human peripheral blood before being stimulated towards differentiation to M1, M2a, M2c or MØ (control) subtypes. **(b)** Heatmap visualization of the TMT proteomic analysis generated from log_2_ fold changes in expression of whole‐cell lysates with subcluster descriptions. Lower expression is represented in blue, while higher expression is in red; false discovery rate set at 5%. Log_2_ fold change values were clustered with hierarchical Euclidean distance clustering. **(c)** Chord plot summarizing the proportion of upregulated and downregulated proteins for each of the M1, M2a and M2c macrophage subtypes with values representing this for each subtype. **(d)** Principal component analysis of the four macrophage subtypes in the total proteomic data set. Dex, dexamethasone; FC, fold change; IFN, interferon; IL, interleukin; NT, not treated; PBMC, peripheral blood mononuclear cell; TNF, tumor necrosis factor.

We next pursued a global overview of the proteomic landscape of different cultured macrophage subtypes through a tandem mass tagging (TMT)–based whole‐cell proteomics approach, comparing polarity states simultaneously. MØ control, M1, M2a and M2c lysates were produced from the same donor material in triplicate (Figure 2a). The log_2_ fold change (log_2_ FC) of protein levels in individual subtypes relative to the originating MØ control population was analyzed. Within the data set, comprising a total of 5435 proteins, all subtypes differ significantly by over 50% of the proteome (Supplementary table 1). This broad variation observed between subtypes is highlighted within the heatmap in Figure 2b. Clear patterns of specific biological regulation are identifiable among this subcluster‐based analysis, such as a strong IFN/cytokine response in M1 macrophages and the upregulation of cell‐matrix adhesion and collagen in M2c macrophages. A filtered data set of differentially expressed proteins (absolute mean log_2_ FC > 1) is provided in Supplementary table 2. These proteins were then processed through the STRING (Search Tool for the Retrieval of Interacting Genes/Proteins) database to create protein–protein interaction networks to facilitate visualization and analysis of the distinct proteomic landscape of each subtype (Supplementary figure 2a–c). The relationships between the three polarized macrophage subtypes can be visualized in the chord plot (Figure 2c), where differentially expressed proteins and their directionality are clustered by co‐occurrence between subtypes. As an additional confirmation of these differential patterns of gene expression regulation, we performed a principal component analysis which identified four distinct clusters, each corresponding to a macrophage subtype with the MØ control central to the analysis (Figure 2d).

**Figure 3:**
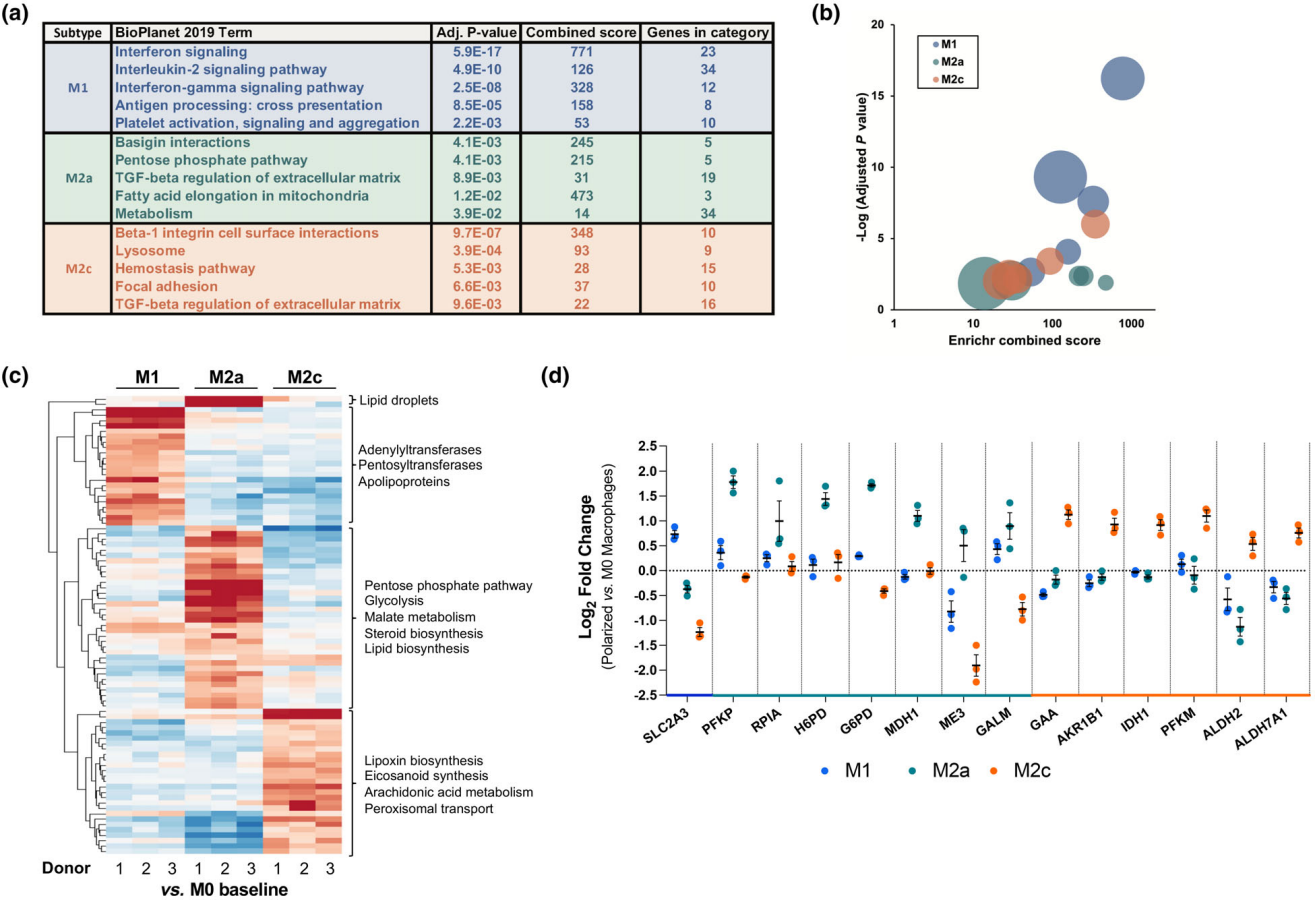
Semiquantitative TMT proteomics elucidates specific protein changes in polarized macrophage populations. **(a)** The top five enriched meta‐categories per macrophage subtype according to the Enrichr BioPlanet 2019 enrichment module. **(b)** Bubble plot from results in **a**. **(c)** Log_2_ fold change heatmap of subset‐specific overexpressed proteins encompassed in the general “Primary Metabolic Process” (totaling 85 proteins, with MØ as a baseline). Gene ontology terms enriched in each protein cluster are displayed to the right. Lower expression is shown in blue and higher expression in red. **(d)** Mean (± standard error of the mean) log_2_ fold change of differentially expressed tricarboxylic acid cycle and pentose phosphate–associated pathway proteins, grouped by polarized macrophage subset specificity. TNF, tumor necrosis factor.

To further dissect the molecular pathways activated upon polarization, a subset‐centered investigation of differentially expressed proteins was pursued through gene set enrichment analysis with the Enrichr BioPlanet 2019 module. This confirmed that M1 macrophages exhibited a heavily proinflammatory profile, as anticipated, while M2a were skewed toward chemokine‐based signaling and M2c possessed a broad regulatory and extracellular matrix–centric profile. A summary table displaying the top five enriched meta‐categories for each macrophage subtype compared with MØ is presented in Figure 3a, with a corresponding bubble plot in Figure 3b (where bubble size denotes the number of genes in the category shown in the table) and Supplementary tables 3–5. Biologically relevant alterations in protein abundance were observed, including the M1 upregulation of proteins with antiviral activity (Mx1 and Mx2^15^ and OAS1‐3^16^). The M2a subtype–upregulated proteins included C‐type lectin family members which mediate immune activity (CLEC4a 5a, 10a and 16a^17^) as well as proteins required for interacting with immune cells including platelets (platelet factor 4^18^) and monocytes and granulocytes (integrin alpha‐M^19^). Conversely, M2c broadly upregulated genes from the collagen family, which in turn were downregulated in the M1 subtype. M2c also upregulated integrin alpha‐V, which mediates interactions with extracellular matrix constituents.^20^, ^21^

Interestingly, both the cluster‐based investigation of differentially expressed proteins (Figure 2b) and the BioPlanet gene set enrichment analysis–based investigation (Figure 3a) also identified an enrichment of pentose phosphate pathways to be particularly specific to the M2a subtype. As changes in metabolic regulation constitute a hallmark of macrophage polarization,^22^ these were investigated through filtering the total whole‐cell data set for metabolism‐related proteins with log_2_ FC > 0.5 among any subset, resulting in a list of 85 proteins with high subset specificity (Figure 3c). Refining these further to include only tricarboxylic acid cycle and pentose phosphate pathway genes as major gene sets in metabolic pathways identified specific upregulation signatures underlying both M2a and M2c subsets separately but not M1 (Figure 3d). Conversely, M1 displayed highly specific upregulation of apolipoproteins, as previously described for IFNγ polarization^23^, ^24^ (Supplementary figure 3).

### Quantitative “surfaceome” analysis further distinguishes between specific macrophage subtypes

**Figure 4:**
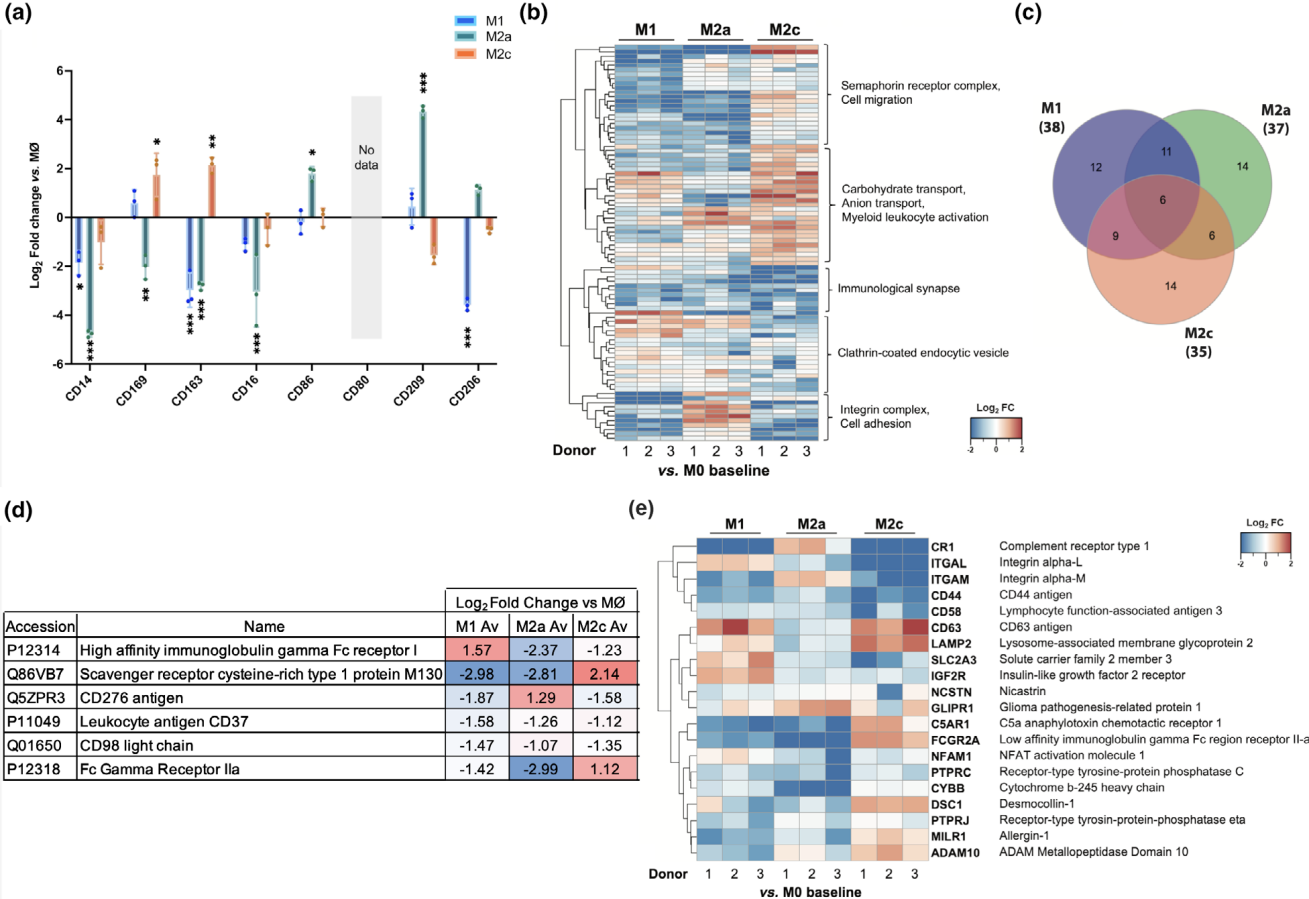
The macrophage “surfaceome” is dynamically altered upon polarization. **(a)** Expression of CD marker proteins (previously assessed in Figure 1b, c) in the tandem mass tagged (TMT) surfaceome analysis after additional filtering for surface proteins using the Cell Surface Protein Atlas *in silico* database^25^ (accessed June 2020). Expression is shown in Log_2_ FC from each subtype against MØ control as a baseline. **(b)** Heatmap visualization of the surfaceome of M1, M2a and M2c subtypes with the MØ control as a baseline. Descriptions of the subclusters are also included. Lower expression is represented in blue, while higher expression is in red. Log_2_ fold change values were clustered *via* hierarchical Euclidean distance clustering. **(c)** Venn diagram of the consistently upregulated and downregulated (log_2_ fold change ≤1 and ≥1) proteins between three subtypes using the MØ control macrophages as a baseline for comparison. **(d)** Table illustrating results from Figure 3b; presented are the proteins either significantly upregulated or downregulated for each of the three subtypes when expressed as a log_2_ fold change (log_2_ fold change ≤ 1 and ≥ 1) against the MØ control. **(e)** Heatmap visualization of the “myeloid cell activation involved in immune response” gene ontology category term in the surface‐filtered TMT data set. *N* = 3 for macrophage subtypes and *N* = 2 for MØ control; false discovery rate was set at 5%.*P < 0.05; **P < 0.01; ***P < 0.001.

We next undertook consecutive surface biotinylation and streptavidin pull downs to capture a snapshot of cell surface proteins expressed across the four cultured macrophage populations. Differential protein expression was evaluated by TMT‐based proteomic analysis, and visualized by comparison with MØ control macrophages (Supplementary figure 4a and table 6). These data sets were filtered to specifically identify cell surface proteins, using the Cell Surface Protein Atlas (CSPA), removing any confounding intracellular proteins.^25^ Expression of key surface markers previously used to identify macrophage subtypes (Figure 1b, c) were assessed utilizing surface proteomics^4^, ^7^, ^9^ (Figure 4a). Differential protein expression was evaluated and visualized, indicating M1 macrophages displayed reduced expression of the key macrophage–identifying surface markers, while M2a had increased expression of CD209 and CD206 compared with MØ unstimulated macrophages, and M2c macrophages upregulated surface levels of CD169 and CD163. This expression profile corroborates markers identified utilizing flow cytometry, highlighting the robustness of this surfaceome data set.

Following CSPA filtering, the surfaceome data set was thus curated to 90 proteins from an original total of 921, and all subsequent analyses were performed with this data set (Supplementary table 7). A new cluster‐based analysis was then performed, further demonstrating broad differences in gene expression between the subtypes with identifiable patterns (Figure 4b). A summary of differential expression of upregulated and downregulated hits between the subtypes is illustrated in Figure 4c. These differentially expressed proteins (*vs*. control MØ) totaled 35 in M2c macrophages, compared with 38 and 37 proteins for the M1 and M2a macrophages, respectively, while only 6 were identified as overlapping between the subtypes (Figure 4c, d). These data also highlight key differences between M2a and M2c subtypes, where these two subtypes shared the least commonly upregulated or downregulated proteins. STRING analysis was also performed for upregulated and downregulated hits (Supplementary figure 4b–g). Evaluation of the Gene Ontology (GO) category “myeloid cell activation in immune response” showed that M2c displayed the broadest response compared with control MØ, significantly upregulating 6 out of 20 hits, namely, CD63 (TIMP‐1 receptor), LAMP2 (lysosome‐associated membrane glycoprotein 2), FCGR2A (low‐affinity immunoglobulin gamma FC region receptor II‐a), DSC1 (desmocollin‐1), MILR1 (allergin‐1) and ADAM10 (Disintegrin and metalloproteinase domain‐containing protein 10) (Figure 4e and Table 1).

**Table 1:** Surfaceome analysis using the Gene Ontology (GO) category “myeloid cell activation in immune response”.

UniProt ID	Gene symbol	Extended gene name	Mean Log_2_ FC M1/M0	Mean Log_2_ FC M2a/M0	Mean Log_2_ FC M2c/M0
P17927	*CR1*	Complement receptor type 1	−3.02	0.58	−3.36
P20701	*ITGAL*	Integrin alpha‐L	0.66	−0.84	−4.02
P11215	*ITGAM*	Integrin alpha‐M	−1.51	0.69	−2.26
P16070	*CD44*	CD44 antigen	−1.31	−0.86	−1.78
P19256	*CD58*	Lymphocyte function‐associated antigen 3	−0.59	−0.73	−1.43
P08962	*CD63*	CD63 antigen	1.44	−0.60	1.48
P13473	*LAMP2*	Lysosome‐associated membrane glycoprotein 2	0.36	−0.64	1.43
P11169	*SLC2A3*	Solute carrier family 2, facilitated glucose transporter member 3	0.89	−0.61	−1.42
P11717	*IGF2R*	Cation‐independent mannose‐6‐phosphate receptor	1.04	−0.48	−0.58
Q92542	*NCSTN*	Nicastrin	−0.46	−0.05	−0.52
P48060	*GLIPR1*	Glioma pathogenesis‐related protein 1	0.07	1.08	0.08
P21730	*C5AR1*	C5a anaphylatoxin chemotactic receptor 1	−1.83	−2.05	0.79
P12318	*FCGR2A*	Low affinity immunoglobulin gamma Fc region receptor II‐a	−1.42	−2.99	1.12
Q8NET5	*NFAM1*	NFAT activation molecule 1	0.22	−1.31	−0.36
P08575	*PTPRC*	Receptor‐type tyrosine‐protein phosphatase C	−0.79	−1.35	−0.56
P04839	*CYBB*	Cytochrome b‐245 heavy chain	−0.49	−2.16	−0.07
Q08554	*DSC1*	Desmocollin‐1	−0.65	−0.91	1.06
Q12913	*PTPRJ*	Receptor‐type tyrosine‐protein phosphatase eta	−1.02	−0.34	−0.16
Q7Z6M3	*MILR1*	Allergin‐1	−1.37	−0.85	0.50
O14672	*ADAM10*	Disintegrin and metalloproteinase domain‐containing protein 10	−1.13	−0.11	0.80

FC, fold change.

### Macrophage protein expression is primed for subtype‐specific functionality

**Figure 5:**
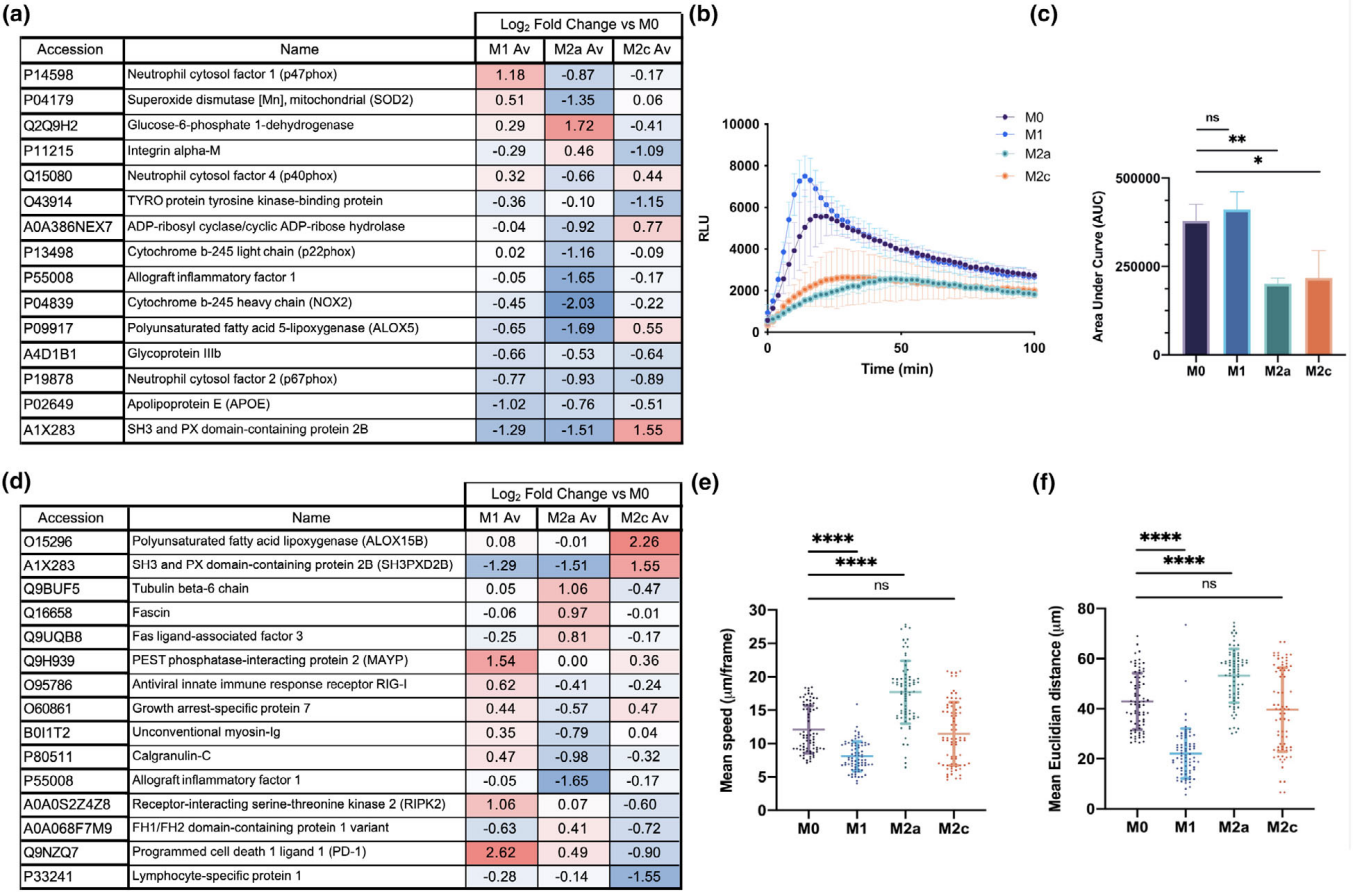
Protein expression of macrophage subtypes is primed for specific functionality. **(a)** Expression of proteins of the “Oxidative stress” GO category term in the gene set enrichment analysis (GSEA)–filtered whole‐cell tandem mass tagged (TMT) data set using the GO Cellular Component 2021 module in Enrichr, filtered for macrophage‐labeled proteins from RNA‐seq data within the ARCHS4 database.^58^ **(b)** Respiratory burst formation in response to phorbol 12‐myristate 13‐acetate (PMA) stimulation measured for each of the four macrophage subtypes for 100 min. Error bars represent the standard deviation. **(c)** Area under the curve analysis for **b**, where a Kruskal–Wallis test (**P < 0.01) followed by Dunn's multiple comparison test was performed (*N* = 3, **P < 0.01 and *P < 0.05). **(d)** Expression of proteins of the “Cytoskeleton” GO category term in the GSEA‐filtered whole‐cell TMT data set using the GO Cellular Component 2021 module in Enrichr, filtered for macrophage‐labeled proteins from RNA‐seq data within the ARCHS4 database.^58^ **(a, d)** Blue denotes decreased expression (log_2_ fold change < 0), red denotes decreased expression (log_2_ fold change > 0) and white denotes no change (log_2_ fold change = 0) in comparison to MØ control. *N* = 3 for macrophage subtypes and *N* = 2 for MØ control; false discovery rate (FDR) was set at 5%. **(e)** Scatter plot with mean and standard deviation of the mean speed of macrophages in each Incucyte imaging field per 20‐min time frame of the four macrophage subtypes. The Kruskal–Wallis test (****P < 0.0001) followed by Dunn's multiple comparison test was performed on 75 fields of view (25 fields of view per donor, *N* = 3, ****P < 0.0001). **(f)** Scatter plot with mean and standard deviation of the mean Euclidean distance macrophages cover in each imaging field per 20‐min time frame of the four macrophage subtypes. The Kruskal–Wallis test (****P < 0.0001) followed by Dunn's multiple comparison test was performed on 75 fields of view (25 fields of view per donor, *N* = 3, ****P < 0.0001). ns, not significant.

We next explored cell functionality, in particular that linked to the expression of specific proteins involved in the motility of macrophage subtypes and the production of reactive oxygen species (ROS) as an indicator of cell activity. The expression of proteins related to ROS production, a hallmark of the macrophage immune response, was extracted with gene set enrichment analysis to identify “oxidative stress” proteins within the total proteome data set, using the GO Cellular Component 2021 module in Enrichr. This analysis detected a total of 34 proteins relating to cytoskeletal function (Supplementary table 8). A comparison of these with the macrophage‐labeled proteins from RNA‐seq data within the ARCHS4 database identified 15 key proteins involved in ROS production (Figure 5a). Interestingly, M1 macrophages upregulated or maintained expression of the most (13 of the 15 identified) ROS‐related proteins (absolute mean log_2_ FC > −1 and < 2) compared with MØ control. M2a and M2c macrophages downregulated six and two proteins compared with MØ control, respectively, with neither subtype upregulating the key ROS‐mediating proteins such as the nicotinamide adenine dinucleotide phosphate (NAPDH) oxidase components p47^phox^ and p40^phox^, or superoxide dismutase 2 (SOD2), in contrast to the M1 subtype.^26^ ROS production was measured in each subtype using a luminol‐amplified chemiluminescence assay. Following stimulation with 100 nM phorbol 12‐myristate 13‐acetate, each generated a respiratory burst in response (Figure 5b, c). IFNγ‐induced M1 macrophages displayed the highest production of ROS as observed previously,^27^, ^28^ with a characteristic initial peak of ROS production observed before a plateau of continuous production. Interestingly, control MØ macrophages also exhibited ROS production levels comparable to M1 macrophages, although to a lesser degree for the initial peak, suggestive of a priming mechanism by IFNγ. Both the M2a and M2c subtypes generated ROS at significantly reduced rates to MØ macrophages (*P* ≤ 0.01 and *P* ≤ 0.05, respectively).

Similarly, gene set enrichment analysis was used to extract all “Cytoskeleton” proteins within the total proteome data set, identifying a total of 85 proteins related to cytoskeletal function (Supplementary table 9), which were again filtered by comparison to the ARCHS4 database to distinguish 15 key proteins involved in macrophage motility (Figure 5d). Expression of tubulin beta 6 chain, a major structural constituent of the cytoskeleton, and the actin bundling protein Fascin^29^ were both upregulated in M2a subtypes, while expression in M1 and M2c did not differ from the MØ control. M2c macrophages upregulated only four of the identified proteins compared with MØ control, although these included SH3PXD2B, an adapter protein involved in actin‐mediated lamellipodia formation,^30^ and ALOX15B, which regulates immune activity.^31^ In M1 cells upregulation of cytoskeletal components involved in proinflammatory immune response was observed, such as the upregulation of RIPK2, required for NOD1‐mediated detection of bacteria,^32^ and programmed cell death protein 1 (PD‐1), which is observed to regulate migration in activated T cells.^33^ The motility of the four subtypes was assessed by using a recently described live‐cell imaging pipeline.^7^, ^13^ Spontaneous motility of each macrophage subtype was observed without the presence of any additional chemotaxis stimuli, which can affect polarization of the cells. Figure 5e demonstrates the significantly reduced (*P* < 0.0001) motility of M1 macrophages and increased motility of M2a macrophages when assessing the mean speed in comparison to MØ control macrophages. M2c macrophages displayed a similar mean speed, albeit with a greater heterogeneity. The mean Euclidean distance, a measure of distance between the start and end of a cell's travel, from each subtype was significantly different (*P* < 0.0001; Figure 5f). Cells in the M1 subtype traveled the least distance compared with the MØ control, while M2a macrophages traveled furthest, and M2c macrophages displayed a similar mean Euclidean distance to that of originating MØ control.

### Macrophage subtypes maintain a set of commonly expressed proteins which facilitate repolarization

**Figure 6:**
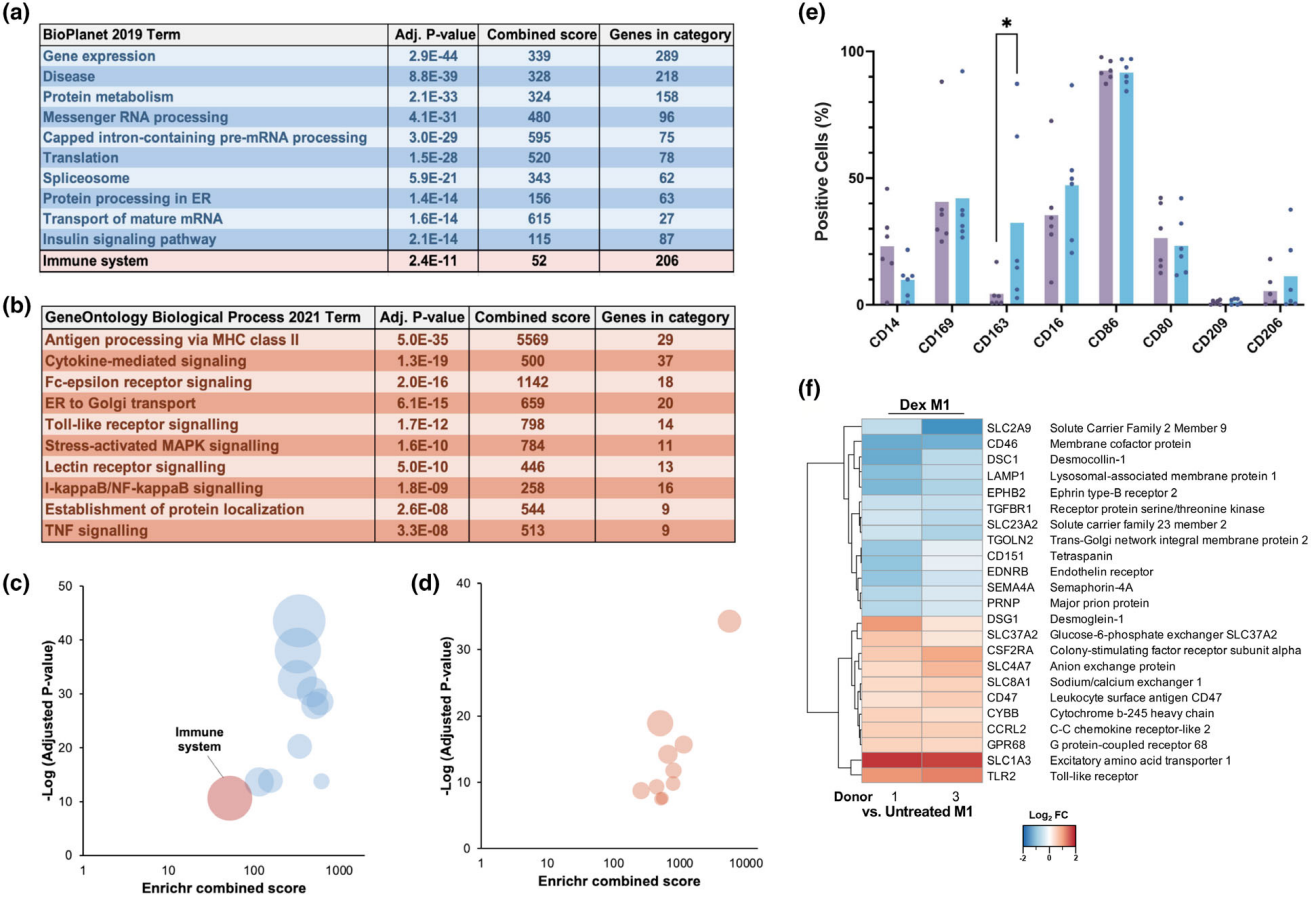
*In vitro* polarized macrophages are able to undergo partial repolarization through the maintenance of a core set of commonly expressed proteins. **(a)** The top 11 enriched meta‐categories per macrophage subtype according to the Enrichr BioPlanet 2019 enrichment module of commonly expressed proteins within the tandem mass tagged proteomic data set generated from log_2_ fold changes in expression of whole‐cell lysates compared with MØ control, where log_2_ fold change is ≥−1 and ≤1. **(b)** The top 10 enriched meta‐categories per macrophage subtype according to the Enrichr BioPlanet 2019 enrichment module of “Immune system” proteins identified in Figure 5a. **(c)** Bubble plot from results in **a**, where red bubbles denote the “Immune system” meta‐category. **(d)** Bubble plot from results in **b**. **(e)** Flow cytometry analysis of dexamethasone treatment for 48 h on M1‐induced macrophages to determine plasticity of inflammatory macrophages. Subtype‐specific markers were analyzed: CD14, CD169, CD163, CD16, CD86, CD80, CD209 and CD206. Percentage positive of population is shown. Bar shows mean, with each point representing individual samples (*N* = 6, ns *P* > 0.05), with a minimum of 10 000 events per sample; significance was tested using a two‐tailed *t‐*test. **(f)** Heatmap visualization of proteomic comparison showing significantly upregulated or downregulated proteins when comparing dexamethasone‐treated samples against M1 as a baseline. *N* = 2 for M1 dexamethasone treated and MØ control; false discovery rate was set at 5%. ns, not significant; *P < 0.05.

The ability to repolarize is a striking hallmark of macrophages, allowing for a broad range of specialized functions and adaptability to the environment. To better understand how macrophages are capable of this swift change in function, we investigated the commonly expressed proteins among the four cultured subtypes. The abundance of 2552 proteins remained fully consistent (absolute mean log_2_ FC > −1 and < 1) across all polarized subtypes and MØ control (Supplementary table 10). A summary table displaying the top enriched meta‐categories for each macrophage subtype within the commonly expressed protein data set is presented in Figure 6a (Supplementary table 11), with a corresponding bubble plot for ease of visualization in Figure 6b. Although this observation is consistent with the common origin of all subtypes from the same macrophage population, beyond the anticipated housekeeping genes, the GO term “immune” was significantly enriched as commonly expressed between the four subtypes. Highlighted within this are the “immune system”–related proteins, totaling 206, which were identified as being maintained across all subtypes (Supplementary table 12). Of these proteins the most abundant categories were composed of proteins associated with antigen processing, cytokine signaling and Fc signaling (Figure 6c, d). The “Immune system” proteins were then processed through the STRING database creating protein–protein interaction networks to facilitate visualization and analysis (Supplementary figure 5). Of note, proteins required for c‐type lectin signaling are maintained across the subtypes, including raf1, which has a well‐defined role in macrophage repolarization and immune function.^34^

### Dexamethasone treatment of proinflammatory M1 macrophages shifts cell polarity toward a deactivated M2 phenotype

Dexamethasone has been widely used as a treatment for a variety of immunopathologies since the 1960s. We therefore utilized this macrophage model system to investigate the effect of dexamethasone on M1 macrophages. Isolated CD14^+^ monocytes were polarized to M1 macrophages using IFNγ before simultaneous treatment with dexamethasone for 48 h to mimic disease pathology in patients and then an analysis was conducted on day 7. Dexamethasone‐treated M1 macrophages had increased levels of CD163 and CD206, albeit neither were found to be significantly increased, both of which are known markers of M2 macrophages.^7^, ^8^ Conversely, the M1 markers CD80 and CD86 remained unchanged with dexamethasone treatment (Figure 6e and Supplementary figure 6a). Treatment with dexamethasone for 24 h displayed a similar, albeit weaker trend than that of the 48‐h treatment (data not shown). In addition, the 48‐h treatment with dexamethasone did not induce a reduction in ROS production, when compared with untreated M1 macrophages (Supplementary figure 6b). To further assess dexamethasone effects on the total proteome, MØ control, M1, M2c and M1 dexamethasone‐treated cells were surface biotinylated and TMT‐labeled. Results were again filtered using the CSPA database, resulting in a final data set of 160 cell surface proteins (Supplementary table 13). Supplementary figure 6c shows a broad comparison of the effects of dexamethasone displayed as log_2_ FC, using MØ as a baseline. Interestingly, only 24 of the 160 surface proteins detected were significantly differentially expressed on comparison of untreated and dexamethasone‐treated M1 cells (Figure 6f, Supplementary figure 4d and Table 2). For example, SLC2A9, CD46 and LAMP‐1 were significantly decreased, whereas SLCA3, TLR2 and CSF2RA were significantly increased upon treatment with dexamethasone, indicating that a 48‐h treatment only partially affects the surface protein abundance of these cells.

**Table 2:** Significantly upregulated or downregulated hits from the surfaceome analysis of dexamethasone treatment, expressed as a log_2_ fold change against M1 samples.

UniProt ID	Gene symbol	Extended gene name	Log_2_ FC DexM1‐A/M1	Log_2_ FC DexM1‐B/M1
P43003	*SLC1A3*	Excitatory amino acid transporter 1	1.79	1.71
O60603	*TLR2*	Toll‐like receptor 2	1.11	1.26
B5M450	*SLC4A7*	Anion exchange protein	0.64	0.97
Q8TED4	*SLC37A2*	Glucose‐6‐phosphate exchanger	1.08	0.36
P32418	*SLC8A1*	Sodium/calcium exchanger 1	0.47	0.83
P42081	*CD86*	CD86 antigen	0.59	0.61
A0A024R6D5	*GPR68*	G protein‐coupled receptor 68	0.56	0.53
Q08722	*CD47*	Leukocyte surface antigen CD47	0.49	0.58
P15509	*CSF2RA*	Granulocyte‐macrophage colony‐stimulating factor receptor subunit alpha	0.71	0.31
P04839	*CYBB*	Cytochrome b‐245 heavy chain	0.39	0.62
O00421	*CCRL2*	C–C chemokine receptor‐like 2	0.57	0.44
O75942	*PRNP*	Major prion protein	−0.71	−0.36
Q02413	*DSG1*	Desmoglein‐1	−0.70	−0.45
Q5T7S2	*TGFBR1*	Receptor protein serine/threonine kinase	−0.56	−0.60
A0A024RCB3	*CD151*	Tetraspanin	−0.93	−0.23
D3DVZ8	*SLC23A2*	Solute carrier family 23 (Nucleobase transporters) member 2	−0.48	−0.69
P24530	*EDNRB*	Endothelin receptor nonselective type	−1.00	−0.21
A0A5H1ZRP2	*TGOLN2*	Trans‐Golgi network integral membrane protein 2	−0.52	−0.78
Q9H3S1	*SEMA4A*	Semaphorin‐4A	−0.96	−0.52
A0A024RDY3	*LAMP1*	Lysosomal‐associated membrane protein 1	−1.05	−0.65
P29323	*EPHB2*	Ephrin type‐B receptor 2	−1.13	−0.77
Q08554	*DSC1*	Desmocollin‐1	−1.24	−0.66
Q9NRM0	*SLC2A9*	Solute carrier family 2 facilitated glucose transporter member 9	−0.63	−1.51
P15529	*CD46*	Membrane cofactor protein	−1.25	−1.19

FC, fold change.

## DISCUSSION

In this study, four macrophage polarization states were produced from a single originating monocyte donor (conducted in triplicate), and these cells were shown to have distinct proteomic landscapes and undergo significant changes in protein expression following specific cytokine induction. This work is therefore reassuring to the field in that despite originating from the same MØ population, there is substantial differential protein abundance changes between subtypes illustrating the dynamic polarization capability of MØ cells. Taken together, these data comprise a list of key potential mediators important for polarization of macrophages, for either macrophage phenotype maintenance in the unchanged protein population or specific polarity changes in the differentially expressed proteins. This is an important data resource which the macrophage community can explore to better understand and manipulate macrophage polarity, to help facilitate their use as future cell therapy treatments, and is an important benchmark comparator for immortalized cell‐derived products.

Our current understanding of macrophage polarity has been expanded beyond cell surface marker identification through several previous proteomic investigations. Prior to our study, the most comprehensive study was conducted by Court and colleagues,^35^ who focused on human *ex vivo* CD14^+^‐derived macrophage polarity in response to environmental oxygen tension. While others have more directly compared dexamethasone stimulation in human macrophages^7^ or the M1 and M2 subtypes in mouse models,^36^ and delved into the surfaceome of polarized monocytes,^37^ no study has compared both whole and surface proteomes and the behavior of multiple human macrophage subtypes generated *ex vivo* from the same originating donor to better understand the changes induced by macrophage plasticity. A comparison of the differentially expressed proteins detected in this study with those identified by Court *et al*.^35^ showed extremely high concordance between both data sets when investigating the three subsets, providing confirmation that the differential expression between the subtypes is reproducible and biologically distinct. Furthermore, our data set is richer, with over fourfold the number of detected proteins as differentially expressed proteins. The increased scope of the integrative proteomic analyses performed in our study not only establishes this study as an unparalleled investigation of macrophage subtypes and polarity *ex vivo*, but also provides additional granularity to the field's understanding of initial M2 subset polarization, cross‐polarization and metabolic specificity through focused analyses of the M2c subtype.

Importantly, through whole‐cell semiquantitative TMT proteomics, we demonstrate significant differential protein expression between macrophage subtypes with consequences to macrophage phenotype and function. Despite being derived from the same originating MØ cell population, the proteome analysis showed that a majority (> 50%) of proteins exhibit differential expression in abundance across the induced subtypes, highlighting the substantial cellular changes macrophages undergo during polarization in this primary cell culture model. We identify specific profiles for the M1 proinflammatory subtype and can distinguish between M2a and M2c subtypes, which skew to chemokine or extracellular matrix–based proteins, respectively. Notably, key differences between M2a and M2c macrophages are distinguished within this data set where M2a and M2c shared the least commonly upregulated or downregulated proteins, and in particular displays highly subset‐specific differences in metabolic regulation, with M2c deviating from previous descriptions of interleukin‐4–stimulated macrophages.^24^ Importantly, this demonstrates that subset polarization even among the M2 spectrum results in a broader scope of biological restructuring than expected, and thus is tantalizing for the design of more specific studies investigating tissue‐resident macrophage biology in humans.

Interestingly, our data indicate the expression of proinflammatory markers in the M2c macrophage surface proteome. For example, complement receptor I (CD35) and integrin alpha‐M (CD11b) were increased in M1 and M2c cells compared with control or M2a subtypes. Thus, although M2c cells express significant differences to M1 in their proteome, they appear to be as activated or primed to activate. Further, within the GO category term “myeloid cell activation involved in immune response,” the M2c subtype displayed the most prominent response, further supporting the proinflammatory priming of this subtype.

Functional studies have also enabled us to discern specific differences between the subtypes. Live cell imaging identified motility differences, with M1 macrophages being significantly less motile than control MØ or M2 subtypes, perhaps because they are potentially awaiting a signal for further activation. The significantly increased speed and distance traveled observed in M2a macrophages in comparison to MØ corroborate findings that alternatively activated macrophages are more motile than control or M1 macrophages.^38^ Furthermore, when dissecting the ROS response between subtypes, we observed significant differences in ROS production between different macrophage subtypes. The response of control MØ was comparable to M1 macrophages, both of which had significantly higher ROS production compared with M2a or M2c. Therefore, ROS production and motility are linked to the polarization state of macrophages and, further still, reflect the position of macrophages on the polarization continuum. Further, dexamethasone treatment of M1 macrophages strongly induced expression of the M2c marker CD163, but did not coincide with a reduction in the M1 marker CD80 expression. These intermediate or overlapping features of macrophage polarity have also been observed by others, although previous focus has been on transcriptomics and hypoxia using a simple M1/M2 model.^9^, ^39^ These data suggest that macrophage polarization is essentially a transient continuum, with cells adaptable and able to move along a sliding scale when necessary. It is important to reiterate that although there are differences between subtypes, they all possess the same core proteome. This provides an explanation for how macrophages enable swift polarity changes along a continuum by maintaining a core set of proteins across all subtypes whose protein expression remain constant while flexing the remainder of the proteome to alter the macrophage characteristics.

In summary, we have for the first time conducted whole‐cell and “surfaceome” comparison of four human primary cultured macrophage subtypes, exploring the significant alterations in total proteome, surface markers and behavior associated with the specific subtypes generated and their roles in immunity. It should be noted that additional cues such as other cell types or specific niche signals may also contribute to aspects of polarity that are not captured in our data sets. Nevertheless, this work confirms that macrophages do not constitute stand‐alone phenotypes but instead represent a continuum of subtypes built on a particular cell theme that are plastic. Importantly, we have dissected the proteomic landscape of the subtypes in relation to their function and we also demonstrate that short‐term exposure with dexamethasone causes M1 cells to partially repolarize, exhibiting features of both M1 and M2c macrophage subtypes. This work therefore highlights the exciting potential of *ex vivo* culture to recapitulate macrophage polarity, and as a future test bed for screening immune modulation–directed drugs or for exploring the control of primary or indeed a comparator for induced pluripotent stem cell–derived macrophages for the generation of sustainable cellular therapeutics.

## METHODS

### Macrophage cell culture

Peripheral blood mononuclear cells were isolated from platelet apheresis blood waste (NHSBT, Bristol, UK) from anonymous healthy donors using experimental protocols approved by Research Ethics Committee (REC 12/SW/0199). Peripheral blood mononuclear cells were separated using a PBMC Spin Medium (pluriSelect Life Science, Leipzig, Germany) as described previously.^40^, ^41^ CD14^+^ cells were isolated from peripheral blood mononuclear cells using CD14^+^ magnetic microbeads (Miltenyi Biotec, Bergisch Gladback, Germany) as per the manufacturer's instructions on day 0.^7^ CD14^+^ cells were cultured at a density of 0.33 × 10^6^ mL^−1^ in Roswell Park Memorial Institute (RPMI) 1640 (Gibco, Thermo Fisher, Waltham, MA, USA) medium supplemented with 10% fetal bovine serum (Gibco, Thermo Fisher, Waltham, MA, USA), 25 ng mL^−1^ macrophage–colony stimulating factor (w/v; Miltenyi Biotec, Bergisch Gladback, Germany) and penicillin/streptomycin at 100 U/0.1 mg per mL^−1^ of media, respectively (w/v; Sigma Aldrich, St. Louis, MO, USA). Cells were cultured for a total of 7 days with macrophage–colony stimulating factor–only for the MØ condition, and with macrophage–colony stimulating factor and the following differentiation cytokines for polarized subtypes: interleukin‐4 at 20 ng mL^−1^ (w/v; BioLegend, San Diego, CA, USA) for M2a, IFNγ at 2.5 ng mL^−1^ (w/v; BioLegend, San Diego, CA, USA) for M1 macrophages and dexamethasone 1 μM (Sigma Aldrich, St. Louis, MO, USA) for M2c. Cells were incubated at 37°C with 5% CO_2_. Macrophages were cultured for a total of 7 days where full media changes were performed two times throughout this period.

### Flow cytometry

The primary antibodies used were CD14 VioBlue (BD Biosciences, Heidelberg, Germany; catalog number 558121), CD16 FITC (Miltenyi Biotec, Bergisch Gladback, Germany; catalog number 130‐113‐392), CD80 FITC (BioLegend, San Diego, CA, USA; catalog number 305205), CD86 PE (BioLegend, San Diego, CA, USA; catalog number 305405), CD163 PE (Miltenyi Biotec, Bergisch Gladback, Germany; catalog number 130‐097628), CD169 APC (Miltenyi Biotec, Bergisch Gladback, Germany; catalog number 130‐098‐643), CD206 APC (BD Biosciences, Germany; catalog number 550889) and CD209 VioBlue (BioLegend, San Diego, CA, USA; catalog number 330102). Antibodies were used as per manufacturer's instructions and incubated for 30 min at 4°C. Data were acquired using a MACSQuant flow cytometer (Miltenyi Biotec, Bergisch Gladback, Germany) and analyzed using FlowJo ™ version 10.7 (BD Biosciences, San Jose, CA, USA).

### Incucyte image acquisition and analysis

For imaging, assays cells were seeded at 0.75 × 10^5^ per well of a CELLSTAR 12 Well Plate on day 6 of culture and left to adhere overnight. The following day media were changed to remove suspension cells and images were captured using the Incucyte SX1 Live‐Cell Analysis System (Essen BioScience, Ann Arbor, MI, USA) using a 20× objective (0.45 NA) to capture 25 imaging fields for each donor every 20 min over a 12‐h period. Videos were composed using the Incucyte software and analyzed using a custom coding program, which automates demands on Fiji (ImageJ, National Institutes of Health, Bethesda, MD, USA), which is known as an imaging “macro.” For MØ, M2a and M2c subtypes individual cells were identified as “cells” and tracked over time. M1 macrophages were observed to cluster during cultures, in which case the clusters were tracked as a single object because of the difficulty of tracking the single cells comprising the cluster. Both “cells” and “clusters” underwent the same analysis stream within the custom Fiji macro. Representative videos from this analysis pipeline can be found at the University of Bristol data repository (https://doi.org/10.5523/bris.11qjhj9l4jpfj2i34kob8d4eug).

### Imaging analysis pipeline

Cells were tracked from phase‐contrast videos using the MIA modular workflow plugin for Fiji.^42^, ^43^, ^44^ First, videos were loaded using a JavaCV‐based plugin for Fiji^45^, ^46^, ^47^ with drift in the input videos corrected using a translation‐only affine transform calculated using Fiji's SIFT feature extraction plugin.^48^, ^49^ For the purpose of cell detection, a copy of the raw video was made and converted to 16‐bit without intensity scaling, then passed through a 2D local variance filter (σ = 5 px) to enhance cells against the relatively homogeneous background. This video was binarized using the Otsu threshold method^50^ and holes in the resulting binary image were filled. The raw video was also sequentially passed through a difference of Gaussian filter (σ = 7 px), intensity inverted and converted to 8‐bit such that the full 8‐bit dynamic range was utilized. Extended intensity minima were identified from this processed image^51^ and used as markers in marker‐controlled watershed segmentation of the first binary image, using the difference of Gaussian image as the intensity landscape. Watershed segmentation was restricted to the contiguous foreground‐labeled regions of the first binary image with areas smaller than 2000 px^2^. Areas larger than this were assumed to correspond to clusters of cells and as such yielded unreliable results from watershed segmentation. Contiguous regions of foreground‐labeled pixels were identified as candidate cell objects using connected components labeling.^51^ Any cells smaller than 200 px^2^ were assumed to correspond to noise, so were discarded from further analysis; those larger than or equal to 200 px^2^ and smaller than 2000 px^2^ were labeled as individual cells and those greater than or equal to 2000 px^2^ were labeled as cell clusters. Individual cells were tracked between video frames based on their centroid coordinates using the TrackMate implementation of the Jaqaman algorithm.^52^, ^53^ Only tracks detected for at least five frames were retained for analysis. Cell clusters were similarly tracked. Finally, motion characteristics for individual cells and cell clusters were calculated and recorded. These data are available at the University of Bristol data repository, data.bris, at https://doi.org/10.5523/bris.11qjhj9l4jpfj2i34kob8d4eug. Video playback is optimal using Fiji (ImageJ) or VLC media player.

### Analysis of ROS

ROS production was examined over time using a luminol‐amplified chemiluminescence assay. Macrophages were prepared and stimulated with phorbol 12‐myristate 13‐acetate as previously described,^54^ and chemiluminescence was recorded for 100 min in 2.5‐min intervals (FLUOstar plate reader, BMG LabTech, Ortenberg, Germany).

### Proteomic experimental design

For the whole‐cell proteomics experiment, a total of three separate donors were used to generate the three macrophage subtypes, M1, M2a and M2c. MØ controls were also included for two of the donors, totaling 11 samples. These two controls were used to enable sample tagging and processing as a single combined experimental scheme. The aforesaid design was similarly followed for the surfaceome experiment. For the dexamethasone treatment surfaceome experiment, three samples were used to generate an MØ control, M1 and M1 dexamethasone‐treated sample; however, one sample did not enable further analysis, and therefore only two samples were taken forward, totaling six samples across the three conditions.

### Surface biotinylation and streptavidin immunoprecipitation

Before the proteomic analysis, surfaceome samples were first washed three times in borate buffer (10 mM boric acid, 154 mM NaCl, 7.2 mM KCl and 1.8 mM CaCl_2_) and then biotinylated using EZ‐Link Sulfo‐NHS‐SS‐Biotin (1 mg mL^−1^; Thermo Scientific, Waltham, MA, USA) on ice for 30 min. Samples were then washed a further time in borate buffer before being washed two times in glycine buffer (0.192 M glycine + 25 mM Tris base). Cells were then lysed in lysis buffer with antiproteases [20 mM Tris–HCl pH 8.0, 137 mM NaCl, 10 mM ethylenediaminetetraacetic acid, 1% NP40 (w/v), 10% glycerol (w/v), 100 mM NaF with the addition of 10 mM Na_3_VO_4_ and the antiproteases 2 mM phenylmethylsulfonyl fluoride and Cocktail V (Calbiochem, used as per the manufacturer's instructions)] for 10 min before removal of nuclear pellets by centrifugation. Cell lysate was then added to 100‐μL streptavidin beads for 1 h at 4°C. Beads were then washed three times in lysis buffer with antiproteases before purging and snap freezing.

### Whole‐cell and surfaceome proteomics

For the surfaceome analysis, streptavidin‐isolated samples were reduced (10 mM tris(2‐carboxyethyl)phospine (TCEP), 55°C for 1 h), alkylated (18.75 mM iodoacetamide, room temperature for 30 min) and then digested from the beads with trypsin (2.5 μg trypsin; 37°C, overnight). For the whole‐cell proteomics, 100 μg of each sample was reduced, alkylated and digested with trypsin as above. The resulting peptides were then labeled with TMT eleven‐plex reagents according to the manufacturer's protocol (Thermo Fisher Scientific, Loughborough, UK) and the labeled samples pooled and desalted using a Sep‐Pak cartridge according to the manufacturer's instructions (Waters, Milford, MA, USA). Eluate from the Sep‐Pak cartridge was evaporated to dryness and resuspended in buffer A (20 mM ammonium hydroxide, pH 10) before fractionation by high pH reversed‐phase chromatography using an Ultimate 3000 liquid chromatography system (Thermo Scientific, Waltham, MA, USA). In brief, the sample was loaded onto an XBridge BEH C_18_ Column (130 Å, 3.5 μm, 2.1 × 150 mm, Waters, UK) in buffer A and peptides were eluted with an increasing gradient of buffer B (20 mM ammonium hydroxide in acetonitrile, pH 10) from 0% to 95% over 60 min. The resulting fractions (4 for the surfaceome analysis and 15 for the whole‐cell analysis) were evaporated to dryness and resuspended in 1% formic acid before analysis by nano‐liquid chromatography tandem mass spectrometry using an Orbitrap Fusion Tribrid mass spectrometer (Thermo Scientific, Waltham, MA, USA).

### Nano‐liquid chromatography mass spectrometry

High pH RP fractions were further fractionated using an Ultimate 3000 nano‐LC system in line with an Orbitrap Fusion Tribrid mass spectrometer (Thermo Scientific, Waltham, MA, USA). In brief, peptides in 1% (vol/vol) formic acid were injected onto an Acclaim PepMap C_18_ nano‐trap column (Thermo Scientific, Waltham, MA, USA). After washing with 0.5% (vol/vol) acetonitrile and 0.1% (vol/vol) formic acid, peptides were resolved on a 250 mm × 75 μm Acclaim PepMap C_18_ reverse phase analytical column (Thermo Scientific, Waltham, MA, USA) over a 150‐min organic gradient with a flow rate of 300 nL min^−1^. Solvent A was 0.1% formic acid and solvent B was aqueous 80% acetonitrile in 0.1% formic acid. Peptides were ionized by nano‐electrospray ionization at 2.0 kV using a stainless‐steel emitter with an internal diameter of 30 μm (Thermo Scientific, Waltham, MA, USA) and a capillary temperature of 275°C. All spectra were acquired using an Orbitrap Fusion Tribrid mass spectrometer controlled by Xcalibur 2.1 software (Thermo Scientific, Waltham, MA, USA) and operated in data‐dependent acquisition mode using an SPS‐MS3 workflow. Fourier transform mass spectrometry S1 spectra were collected at a resolution of 120 000, with an automatic gain control target of 200 000 and a maximum injection time of 50 ms. Precursors were filtered with an intensity threshold of 5000, according to charge state (to include charge states 2–7) and with monoisotopic peak determination set to peptide. Previously interrogated precursors were excluded using a dynamic window (60 s ± 10 ppm). The second mass spectrometer (MS2) precursors were isolated with a quadrupole isolation window of 1.2 *m/z*. The ion trap mass spectroscopy 2 (ITMS2) spectra were collected with an automatic gain control target of 10 000, maximum injection time of 70 ms and CID collision energy of 35%. For Fourier transform mass spectrometry S3 analysis, the Orbitrap was operated at 50 000 resolution with an automatic gain control target of 50 000 and a maximum injection time of 105 ms. Precursors were fragmented by high‐energy collision dissociation at a normalized collision energy of 60% to ensure maximal TMT reporter ion yield. Synchronous precursor selection was enabled to include up to 10 MS2 fragment ions in the Fourier transform mass spectrometry S3 scan.

### Proteomic data analysis

The raw data files were processed and quantified using Proteome Discoverer software version 2.1 (Thermo Scientific, Waltham, MA, USA) and searched against the UniProt Human database (downloaded March 2020: 165 104 entries) using the SEQUEST HT algorithm. Peptide precursor mass tolerance was set at 10 ppm, and tandem mass spectrometry tolerance was set at 0.6 Da. Search criteria included oxidation of methionine (+15.995 Da), acetylation of the protein N terminus (+42.011 Da) and methionine loss plus acetylation of the protein N terminus (−89.03 Da) as variable modifications and carbamidomethylation of cysteine (+57.021 Da) and the addition of the TMT mass tag (+229.163 Da) to peptide N termini and lysine as fixed modifications. Searches were performed with full tryptic digestion and a maximum of two missed cleavages were allowed. The reverse database search option was enabled and all data were filtered to satisfy a 5% false discovery rate.

The resulting protein abundance data were normalized against the total abundance of each respective sample. Subsequently, the abundance of each protein from the samples of interest was divided by the average abundance of the respective protein in the two control samples to generate FC data, and log transformed to generate log_2_ FC values. As the final preprocessing step, median centering was performed on the log‐transformed data. For the curated surfaceome data set, the total total dataset was filtered through use of the *in silico* human surfaceome database to generate a cell surface–specific data set.^28^ Log_2_ FC values were clustered *via* hierarchical Euclidean distance clustering and visualized with the R ‘pheatmap’ package (R Foundation, Vienna, Austria). GO, Kyoto Encyclopedia of Genes and Genomes (KEGG) pathway and network analysis of differentially expressed genes were performed through EnrichR^55^ and the STRING database.^56^ Published proteomics data have been deposited to the ProteomeXchange Consortium *via* PRIDE (EMBL‐EBI, Cambridge, UK)^57^ with the data set identifiers PXD032801, PXD032823 and PXD032967. The Supplementary tables of these proteomic data sets are available at the University of Bristol data repository: https://doi.org/10.5523/bris.220i0rql3u6929ay07x99u95e.

### Statistical analysis

Where appropriate, statistical analysis was used to determine statistical significance and stated within the relevant figure captions. In summary, statistical analyses and generation of graphics were performed using GraphPad Prism 9 (version 9.1.0). Details of statistical tests used and numbers of independent experiments are indicated in the figure captions. Standard deviation is shown where applicable. Data were confirmed to be either normally distributed (a = 0.05) or non‐normally distributed *via* the D'Agostino–Pearson omnibus test or the Shapiro–Wilk test prior to further comparisons. When the data were normally distributed, an unpaired two‐sided *t‐*test was used for groups of two sample types, and an ordinary one‐way ANOVA with Dunnet's multiple comparison *post hoc* test was used to compare groups of more than two sample types comparing every mean with the control mean. Alternatively, an ordinary one‐way ANOVA was followed by a Tukey test to compare every mean with every other mean. For non‐normally distributed data, the unpaired two‐sided Mann–Whitney *U*‐test was used for groups of two and the Kruskal–Wallis test with Dunn's multiple comparison *post hoc* test was used to compare groups of more than two sample types comparing every mean with the control mean. For results presented as a percentage of the control, a single sample *t‐*test and the Wilcoxon test were used. Statistical significance is indicated on graphs using standard conventions, as follows: nonsignificant (ns), *P < 0.05; **P < 0.01; ***P < 0.001; ****P < 0.0001.

## AUTHOR CONTRIBUTIONS

**Tiah CL Oates:** Conceptualization; data curation; formal analysis; investigation; methodology; project administration; resources; software; validation; visualization; writing – original draft; writing – review and editing. **Pedro L Moura:** Data curation; formal analysis; investigation; methodology; software; validation; visualization; writing – original draft; writing – review and editing. **Stephen Cross:** Data curation; formal analysis; investigation; methodology; software; writing – original draft; writing – review and editing. **Kiren Roberts:** Data curation; formal analysis; investigation; methodology; writing – original draft; writing – review and editing. **Holly E Baum:** Data curation; formal analysis; investigation; methodology; writing – original draft; writing – review and editing. **Katy L Haydn‐Smith:** Data curation; formal analysis; investigation; methodology; writing – original draft; writing – review and editing. **Mariangela C Wilson:** Data curation; investigation; methodology; writing – original draft; writing – review and editing. **Kate J Heesom:** Data curation; formal analysis; investigation; methodology; writing – original draft; writing – review and editing. **Charlotte E Severn:** Conceptualization; data curation; formal analysis; investigation; methodology; resources; software; supervision; validation; visualization; writing – original draft; writing – review and editing. **Ash M Toye:** Conceptualization; funding acquisition; investigation; methodology; supervision; writing – original draft; writing – review and editing.

## CONFLICT OF INTEREST

The authors declare that the research was conducted in the absence of any commercial or financial relationships that could be construed as a potential conflict of interest. The views expressed are those of the authors and not necessarily those of the National Health Service, NIHR or the Department of Health and Social Care.

## FUNDING

This work was funded by grants from the NIHR Blood and Transplant Research Unit in red cell products (IS‐BTU‐1214‐10032), NHS Blood and Transplant (NHSBT) R&D (WP15‐05) and a Wellcome Trust PhD Studentship to TCLO (8043 WT 108907/Z/15/Z Dynamic Cell), PLM was supported by a Cancerfonden (Swedish Cancer Society) postdoctoral grant (21 0340 PT) and the work by HEB was supported by the Elizabeth Blackwell Institute (University of Bristol) with funding from the University's alumni and friends.

## Supporting information


Supplementary figure 1
Supplementary figure 2
Supplementary figure 3
Supplementary figure 4
Supplementary figure 5
Supplementary figure 6

Supplementary table 1
Supplementary table 2
Supplementary table 3
Supplementary table 4
Supplementary table 5
Supplementary table 6
Supplementary table 7
Supplementary table 8
Supplementary table 9
Supplementary table 10
Supplementary table 11
Supplementary table 12
Supplementary table 13

## Data Availability

The data that support the findings of this study are openly available at the University of Bristol data repository. Supplementary tables of these proteomic data sets are available at https://doi.org/10.5523/bris.220i0rql3u6929ay07x99u95e. For the Incucyte work, data are available at https://doi.org/10.5523/bris.11qjhj9l4jpfj2i34kob8d4eug.
