# Supplementary material for: Defining the proteomic landscape of cultured macrophages and their polarization continuum
Source: Immunol Cell Biol. 2023 Sep 11;101(10):947–63. doi: 10.1111/imcb.12687 (PMC10953363; doi:10.1111/imcb.12687)
Supplement: Supplementary file 1 — Supplementary figure 1 Supplementary figure 2 Supplementary figure 3 Supplementary figure 4 Supplementary figure 5 Supplementary figure 6 [file IMCB-101-947-s002.pdf]

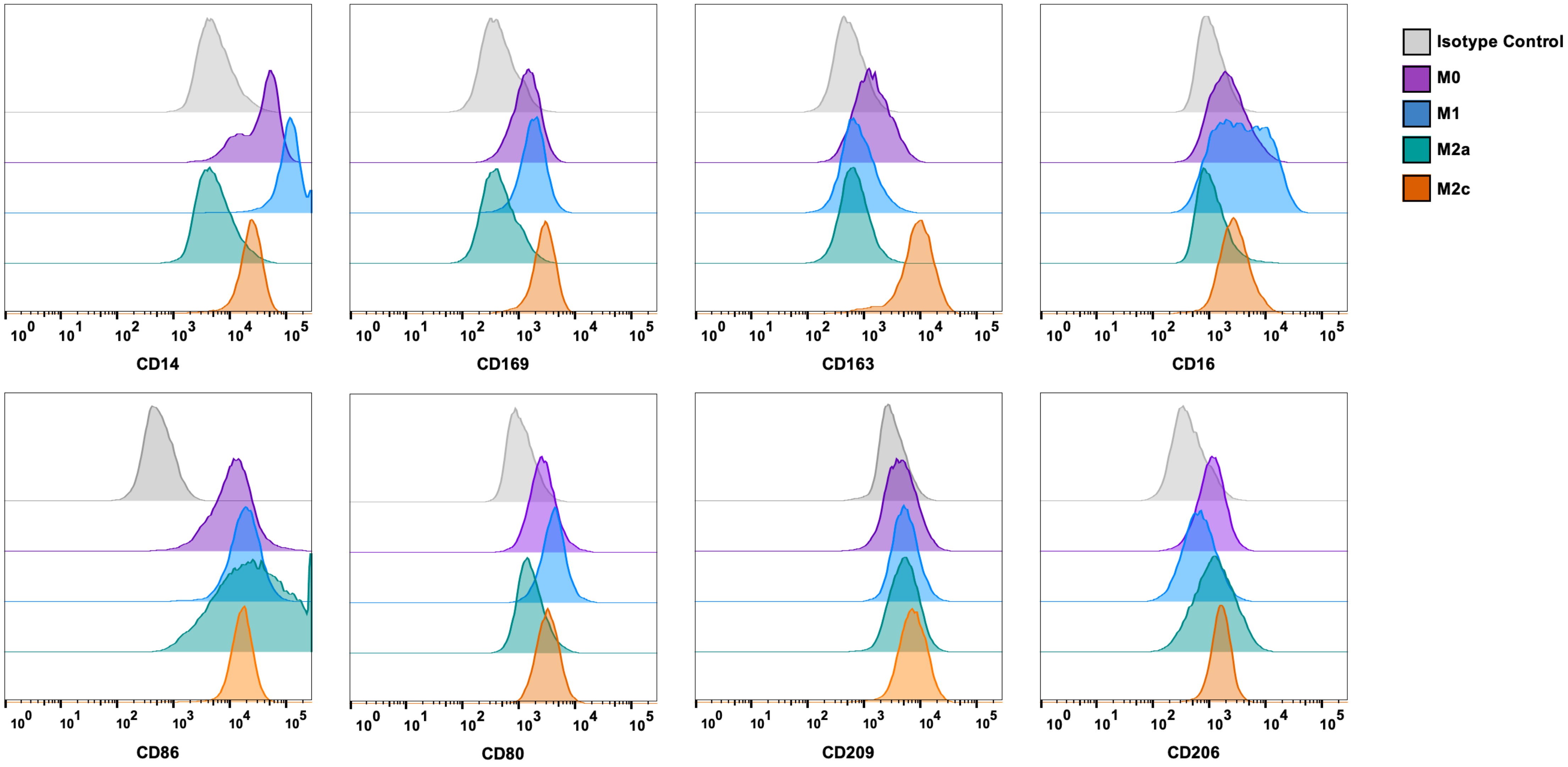

Supplementary figure 1: flow cytometry analysis of cell surface markers. Modal histograms illustrating the expression of CD14 (top left), CD169 (top second left), CD163 (bottom second right), CD16 (bottom right), CD86 (bottom left), CD80 (bottom second left), CD209 (bottom second right), CD206 (bottom right) for populations of macrophages M0 (purple), M1 (blue), M2a (green), and M2c (orange) compared to the isotype control (grey). Single donor shown representative of N=3 with a minimum of 10,000 events per sample.

(a)

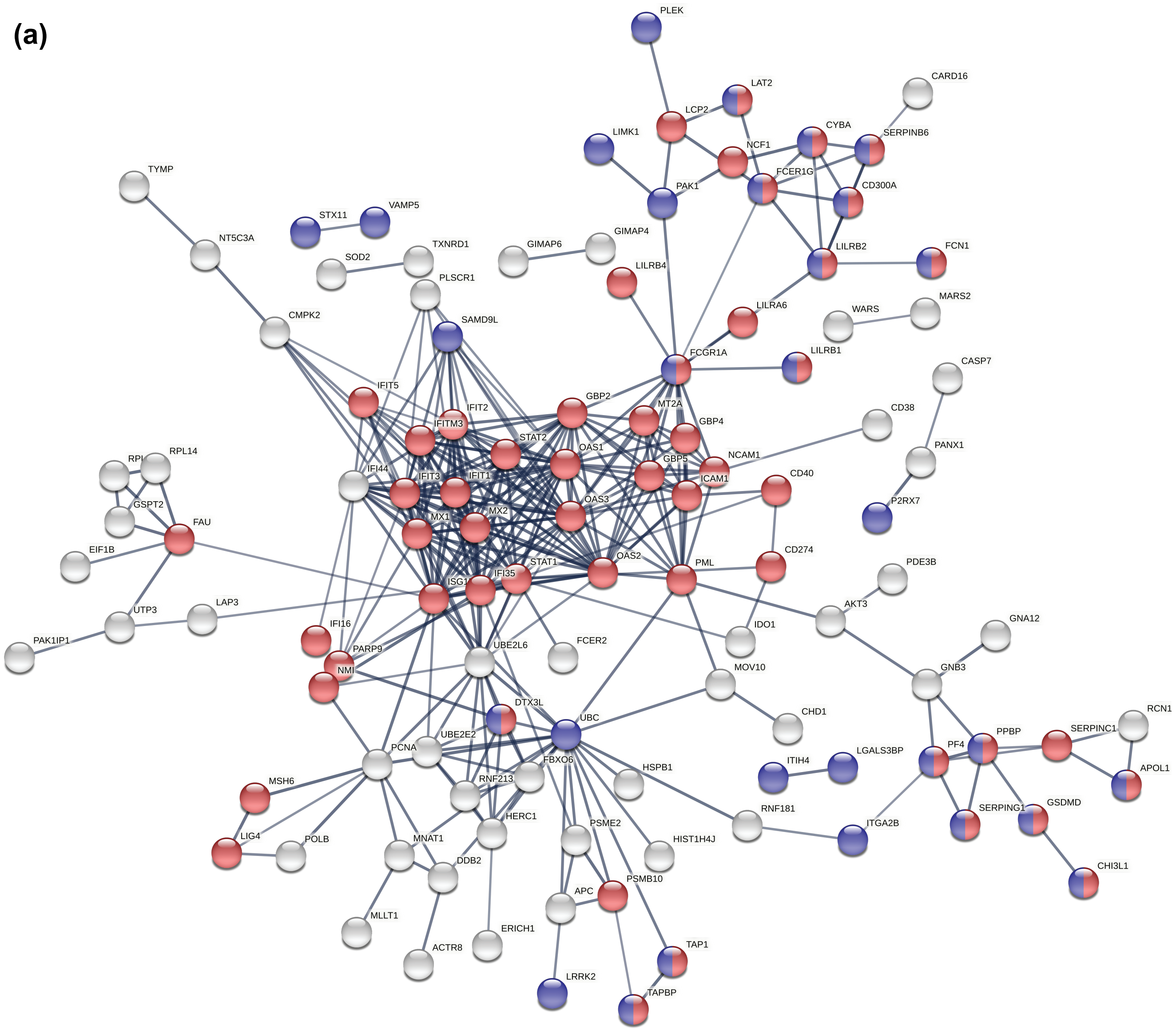

Supplementary figure 2: Network analysis of whole-cell proteomic data. (a) Network of M1 upregulated proteins where nodes in red denote the gene ontology (GO) term immune response and those in blue vesicle transport.

(b) (c)

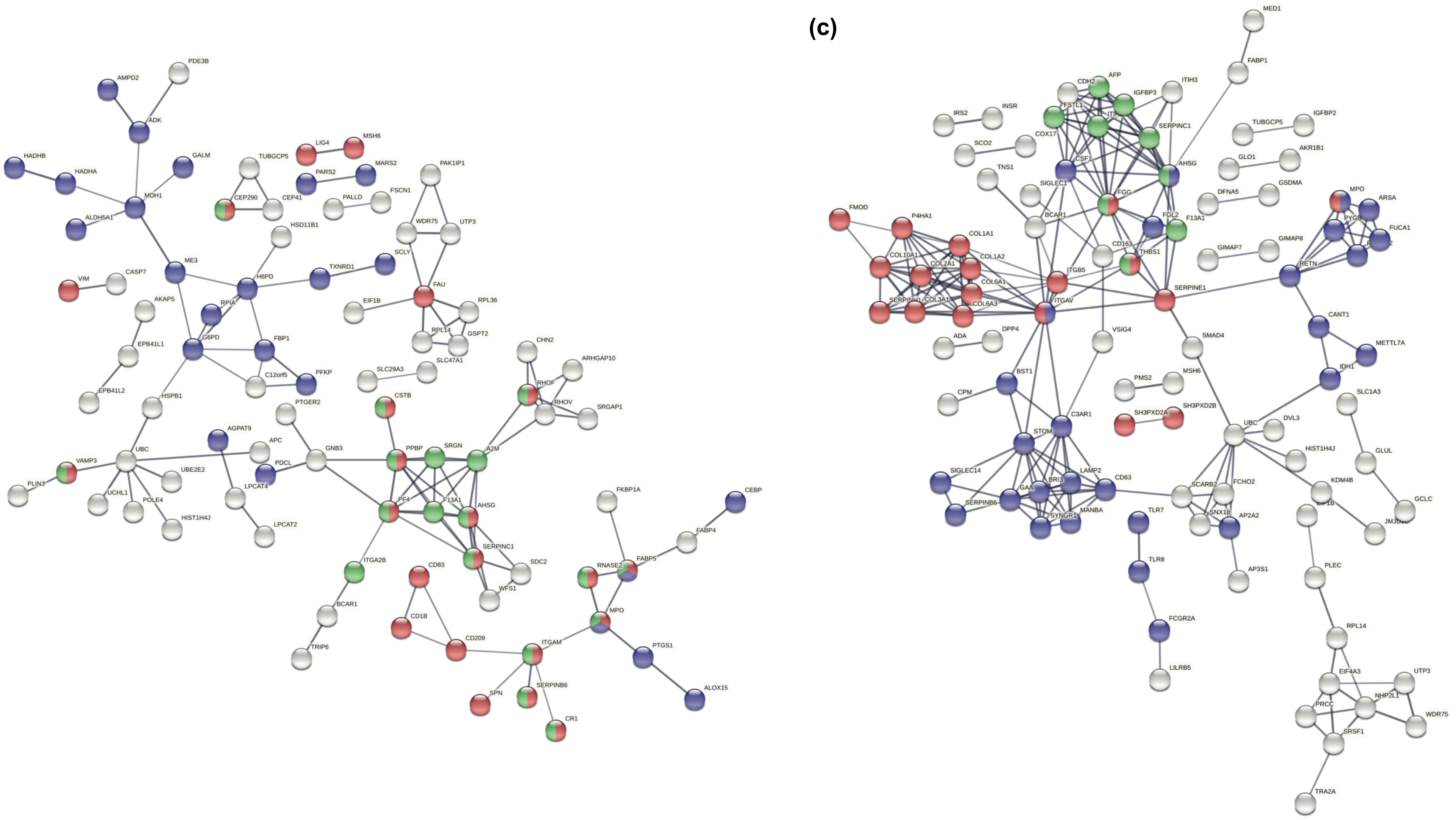

Supplementary figure 2 continued: Network analysis of whole-cell proteomic data. (b) Upregulated proteins for M2a macrophages where red nodes represent the GO term immune response, green secretion, and blue metabolism. **(c)** M2c upregulated proteins from the following GO terms, where red nodes are extracellular proteins, blue for myeloid leukocyte activation and green for IGF transport and metabolism.

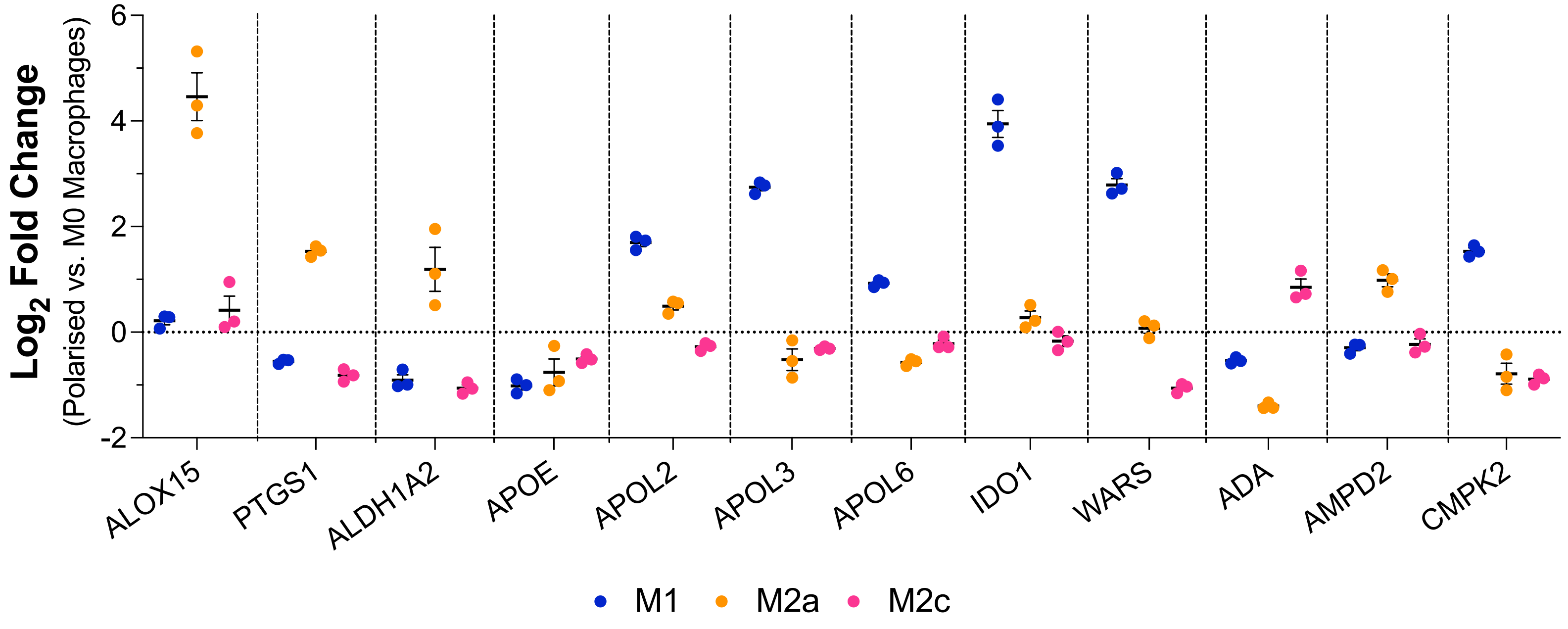

Supplementary figure 3: Expression of metabolism-associated proteins. Mean (± SEM) Log_2FC of metabolism-associated proteins indicated as differentially expressed in polarized M1 and/or M2 macrophages in Murugesan G et al. 2022.

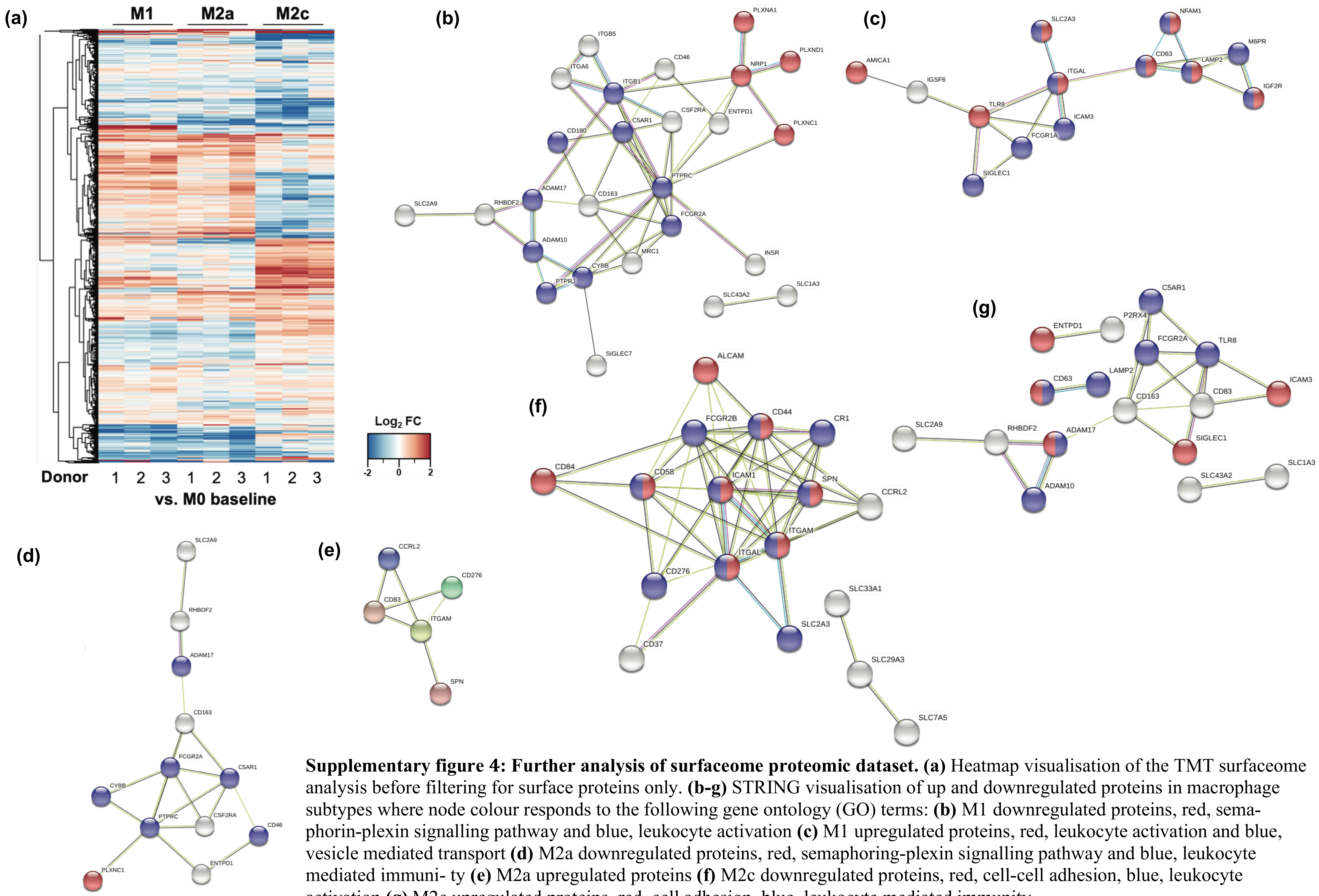

Supplementary figure 4: Further analysis of surfaceome proteomic dataset. (a) Heatmap visualisation of the TMT surfaceome analysis before filtering for surface proteins only. **(b-g)** STRING visualisation of up and downregulated proteins in macrophage subtypes where node colour responds to the following gene ontology (GO) terms: **(b)** M1 downregulated proteins, red, semaphorin-plexin signalling pathway and blue, leukocyte activation **(c)** M1 upregulated proteins, red, leukocyte activation and blue, vesicle mediated transport **(d)** M2a downregulated proteins, red, semaphoring-plexin signalling pathway and blue, leukocyte mediated immuni- ty **(e)** M2a upregulated proteins **(f)** M2c downregulated proteins, red, cell-cell adhesion, blue, leukocyte activation **(g)** M2c upregulated proteins, red, cell adhesion, blue, leukocyte mediated immunity.

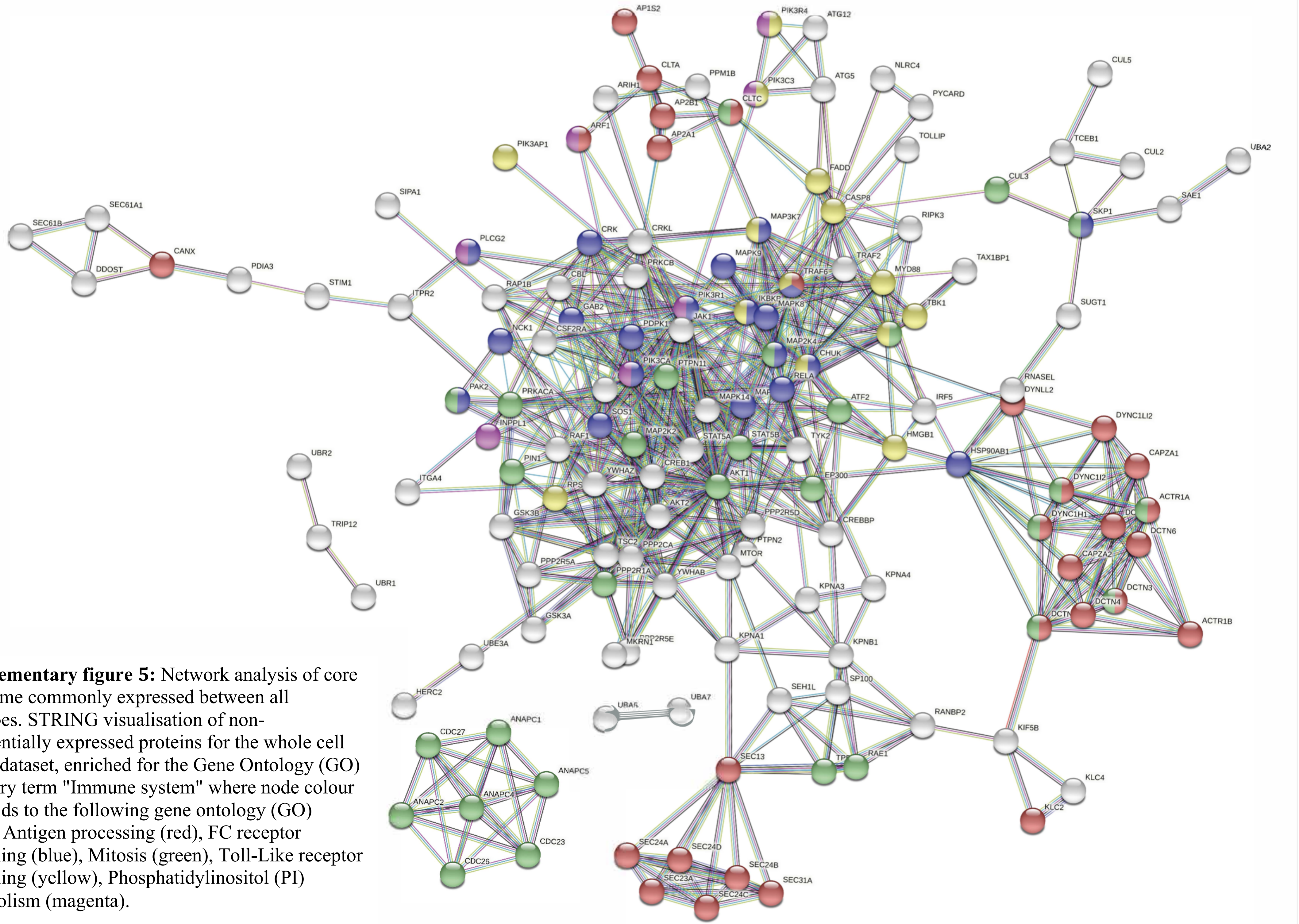

Supplementary figure 5: Network analysis of core proteome commonly expressed between all subtypes. STRING visualisation of non-differentially expressed proteins for the whole cell lysate dataset, enriched for the Gene Ontology (GO) category term "Immune system" where node colour responds to the following gene ontology (GO) terms: Antigen processing (red), FC receptor signalling (blue), Mitosis (green), Toll-Like receptor signalling (yellow), Phosphatidylinositol (PI) metabolism (magenta).

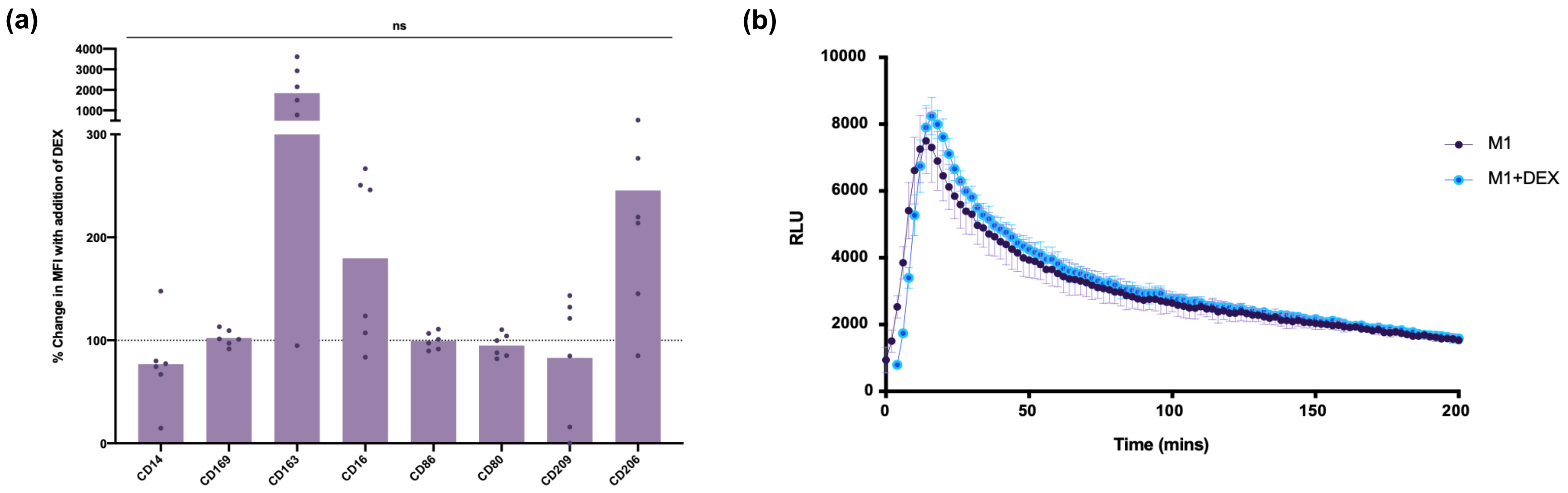

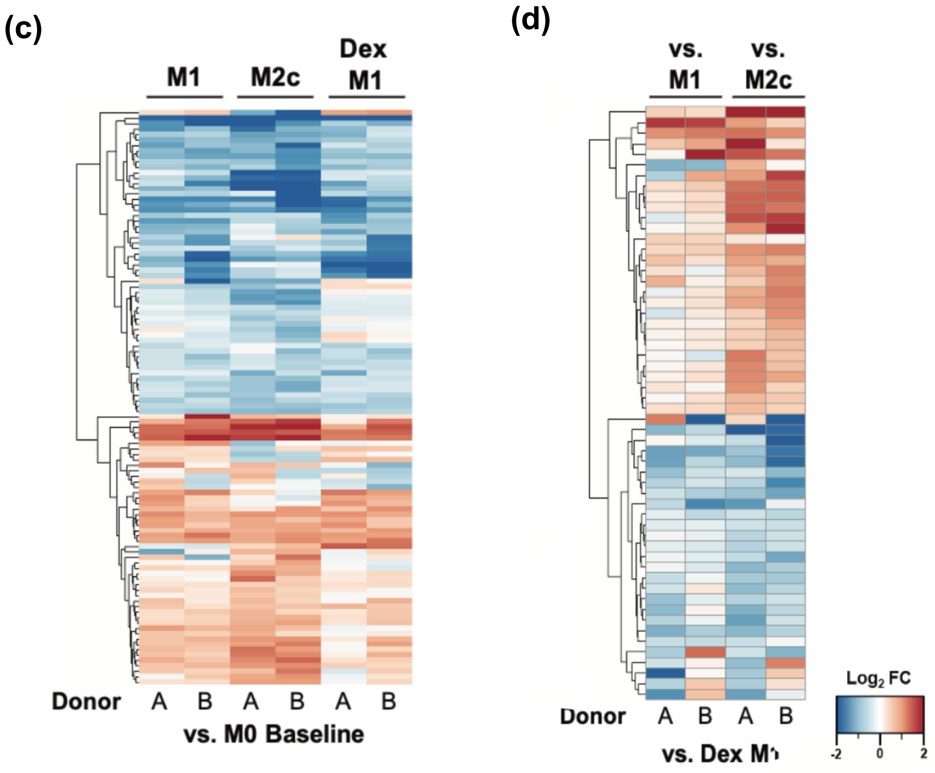

Supplementary figure 6: Differential expression of surface markers in M1 macrophages after 48 hours treatment with dexamethasone. (a) Median fluorescence intensity for flow cytometry presented in Figure 6a. Data presented as a percentage change upon addition of dexamethasone. Dotted line depicts the 100% and subsequently no change in expression. Bar shows mean with each point representation individual samples (N=6, ns $P \geq 0.05$), significance tested using a one sample *t*-test and Wilcoxon test. **(b)** Comparison of respiratory burst formation in M1 macrophages and dexamethasone treated M1 macrophages when stimulated with PMA, N=3. **(c)** Heatmap visualisation of TMT surfaceome analysis filtered for surface proteins generated from log2 fold changes in expression in M1, M2c and M1 dexamethasone treated samples with the MØ control as a baseline. Lower expression represented in blue whilst red denotes higher expression. FDR set at 5%. **(d)** Heatmap visualisation of differential expression generated from $\log_2$ fold changes in expression of surface markers in M1 and M2c subtypes when using M1 dexamethasone macrophages as a baseline. For **(c)** and **(d)** Lower expression represented in blue whilst red denotes higher expression. FDR set at 5 %.
